# Supplementary material for: SH3BP4 Regulates Intestinal Stem Cells and Tumorigenesis by Modulating β-Catenin Nuclear Localization
Source: Cell Rep. 2019 Feb 26;26(9):2266–2273.e4. doi: 10.1016/j.celrep.2019.01.110 (PMC6391711; doi:10.1016/j.celrep.2019.01.110)
Supplement: Document S1. Figures S1–S5 and Table S1 [file mmc1.pdf]

Cell Reports, Volume *26*

Supplemental Information

SH3BP4 Regulates Intestinal Stem Cells and Tumorigenesis by Modulating β-Catenin Nuclear Localization

Pedro Antas, Laura Novellasdemunt, Anna Kucharska, Isobel Massie, Joana Carvalho, Dahmane Oukrif, Emma Nye, Marco Novelli, and Vivian S.W. Li

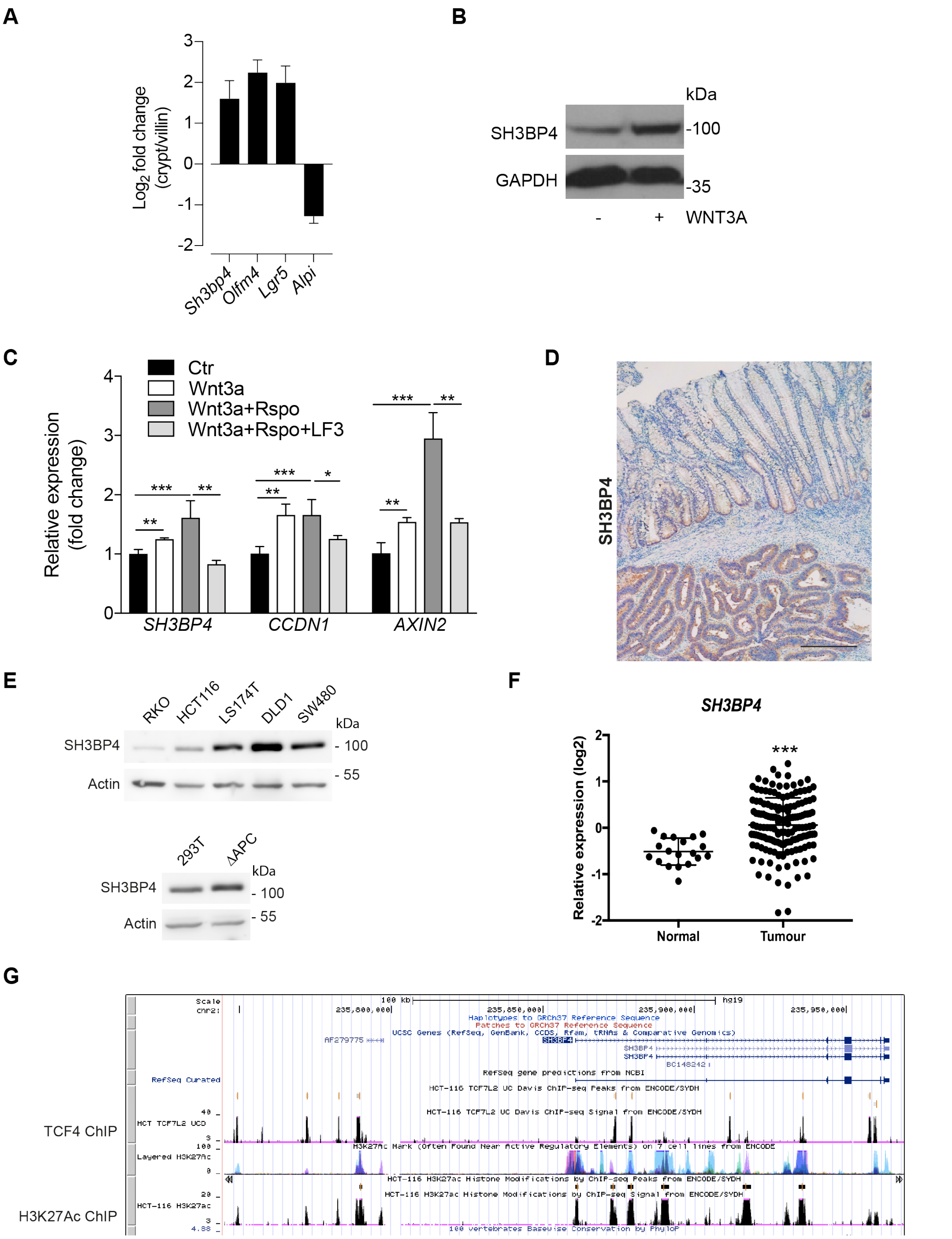

Figure S1. SH3BP4 is expressed at the crypt and is modulated by Wnt, Related to Figure 1.
(A) RT-qPCR analysis of the indicated genes in crypt versus villus fractions. Data represent average ±SD from 3 independent experiments. (B) Western blot analysis of SH3BP4 protein levels in HEK293T cells stimulated with WNT3A condition media for 16hr (n=3). (C) RT-qPCR of *SH3BP4* and Wnt target genes *AXIN2*, *CCND1* in the indicated treatment conditions (16hr). Expression data are presented as fold induction ±SD normalised to β-actin (n=3). (D) Representative immunohistochemistry staining of SH3BP4 in human CRC tissue. Scale bar, 100 μm. (E) Western blot analysis showing SH3BP4 expression in different colorectal cancer cell lines (upper), and in HEK293T and ΔAPC cells (APC4 cells from (Novellasdemunt et al., 2017)) (n=3). (F) mRNA expression analysis of SH3BP4 in 173 CRC patients (data obtained from the Cancer Genome Atlas) (TCGA, 2012). (G) TCF4-binding sites are found upstream and throughout the *SH3BP4* gene locus, and are co-localized with the H3K27Ac peaks. Data obtained from ENCODE where TCF4 ChIP-seq was performed in HCT116 cells (GSM782123) (Consortium, 2012).

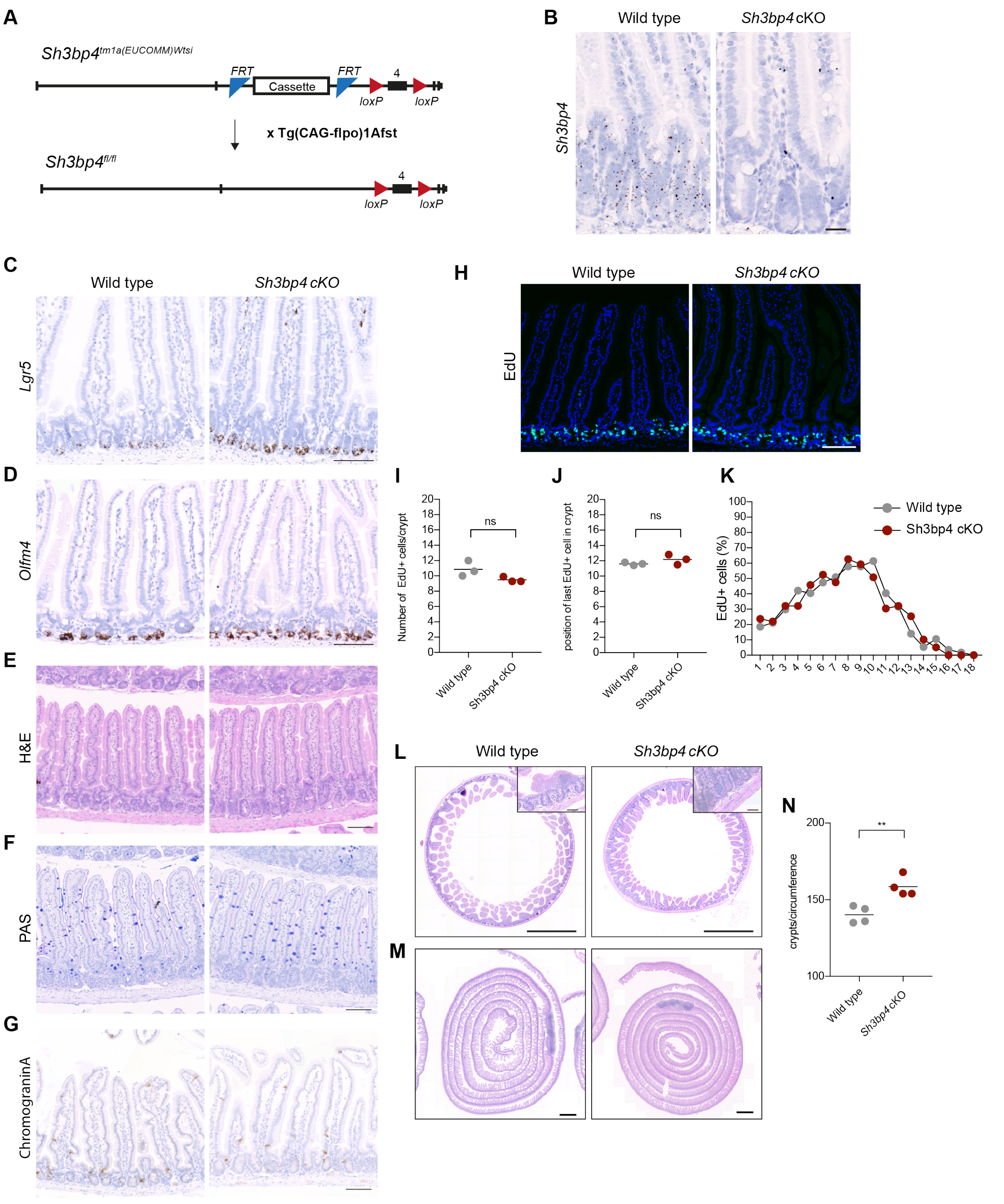

Figure S2. Characterization of *Sh3bp4* cKO intestine 3 months (A-B) or 25 days (C-K) after deletion, Related to Figure 2. (A) Schematic overview of Sh3bp4 targeting strategy. *Sh3bp4*$^{tm1a(EUCOMM)Wtsi}$ mouse was first crossed to Tg(CAG-Flpo)1Afst strain to remove the lacZ-neomycin cassette. This results in the conditional *Sh3bp4*$^{fl/fl}$ strain flanking exon 4. (B) RNAScope ISH showing efficient Cre-mediated *Sh3bp4* deletion in the intestine after tamoxifen induction. Scale bar, 100 µm. (C-D) Representative image of RNAscope ISH of ISC marker, *Lgr5* (C) and *Olfm4* (D), 3 months after *Sh3bp4* loss. (E-G) Tissues stained with H&E (E), periodic acid Schiff (PAS, goblet cells) (F), and chromogranin A (enteroendocrine cells) (G) showed no difference between wild-type and *Sh3bp4* cKO intestine. Scale bar, 100 µm. (H) Immunofluorescent staining of EdU (green) and DAPI (blue). (I-K) Quantitation of EdU+ proliferating cells shows no differences in number of EdU^{+} cells per crypt (I), nor in the height of the proliferative zone (J), nor in the distribution of the proliferative cells along the crypt-villus axis (K). (L-M) H&E staining of wild-type and *Sh3bp4* cKO intestine in transversal (L) and frontal (M) intestinal sections showing more compact crypt-villus units in *Sh3bp4* cKO compared to wild-type. Scale bar, 1mm; inset 100 µm. (N) Quantitation of number of crypts per circumference in the small intestine. Each dot represents the average number of crypts counted in 3 different transversal cuts of the jejunum per mouse. n=4 animals/group. **P≤ 0.01.

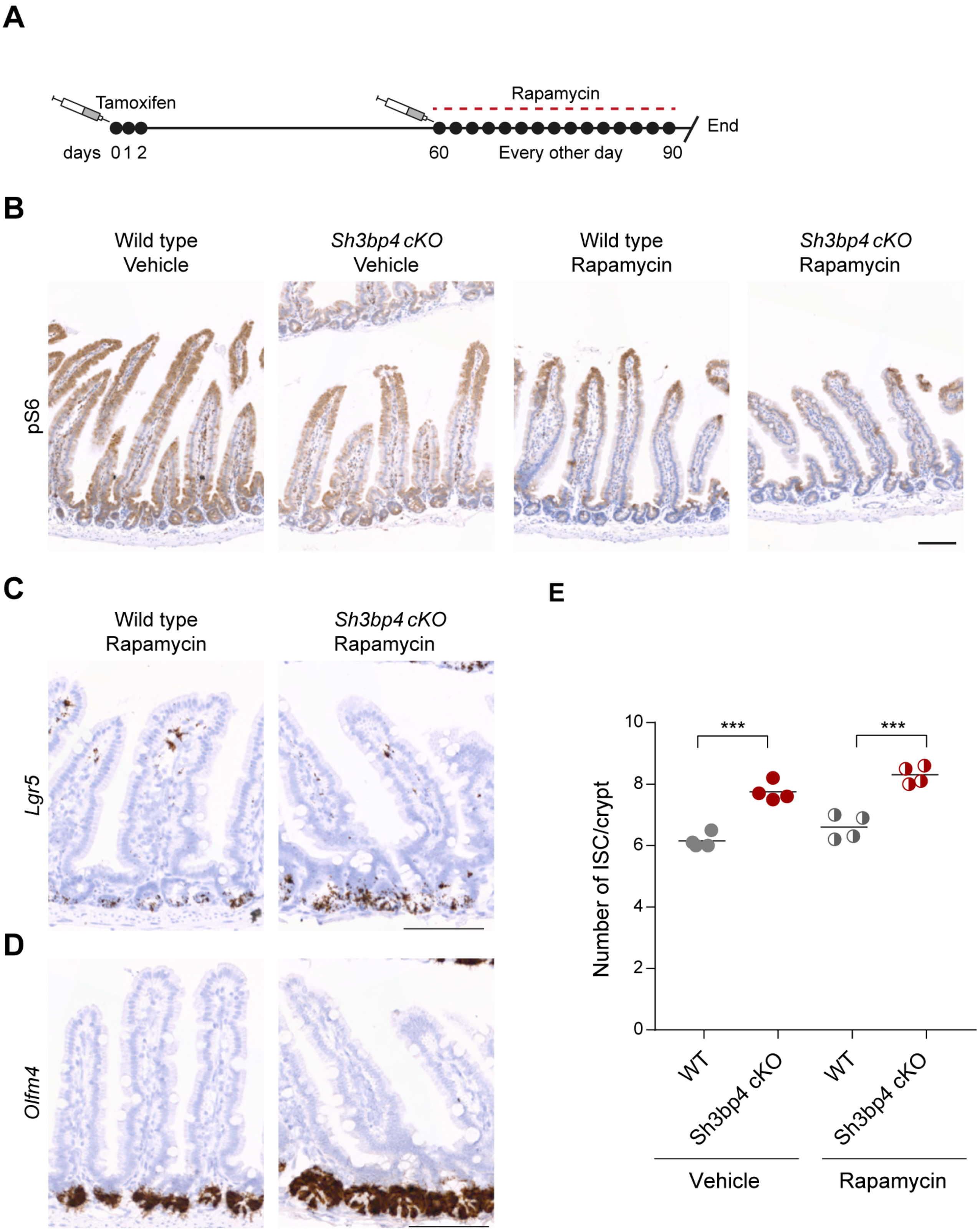

Figure S3. Increased number of stem cells in *Sh3bp4* cKO mice is independent of mTOR pathway, Related to Figure 2. (A) Schematic representation of rapamycin treatment strategy. *Sh3bp4* deletion was induced at 6 weeks-old by tamoxifen administration, and rapamycin treatment started 2 months after with intraperitoneal injections every other day for a month, after which mice were sampled. (B) Representative immunohistochemistry of phospho-RSP6 (pS6) showing reduction of RSP6 phosphorylation in animals treated with rapamycin for a month. (C-D) Representative images of RNAscope ISH of the ISC marker *Lgr5* (C) and *Olfm4* (D). Scale bar, 100µm. (E) Quantitation of number of *Olfm4*+ ISCs per crypt shows that mTOR inhibition has no effect on the increased number of ISCs observed in *Sh3bp4* cKO compared to wild-type. Each dot represents the average number of *Olfm4*+ cells per crypt per mouse (determined from at least 30 crypts analyzed). n=4 animals/group.

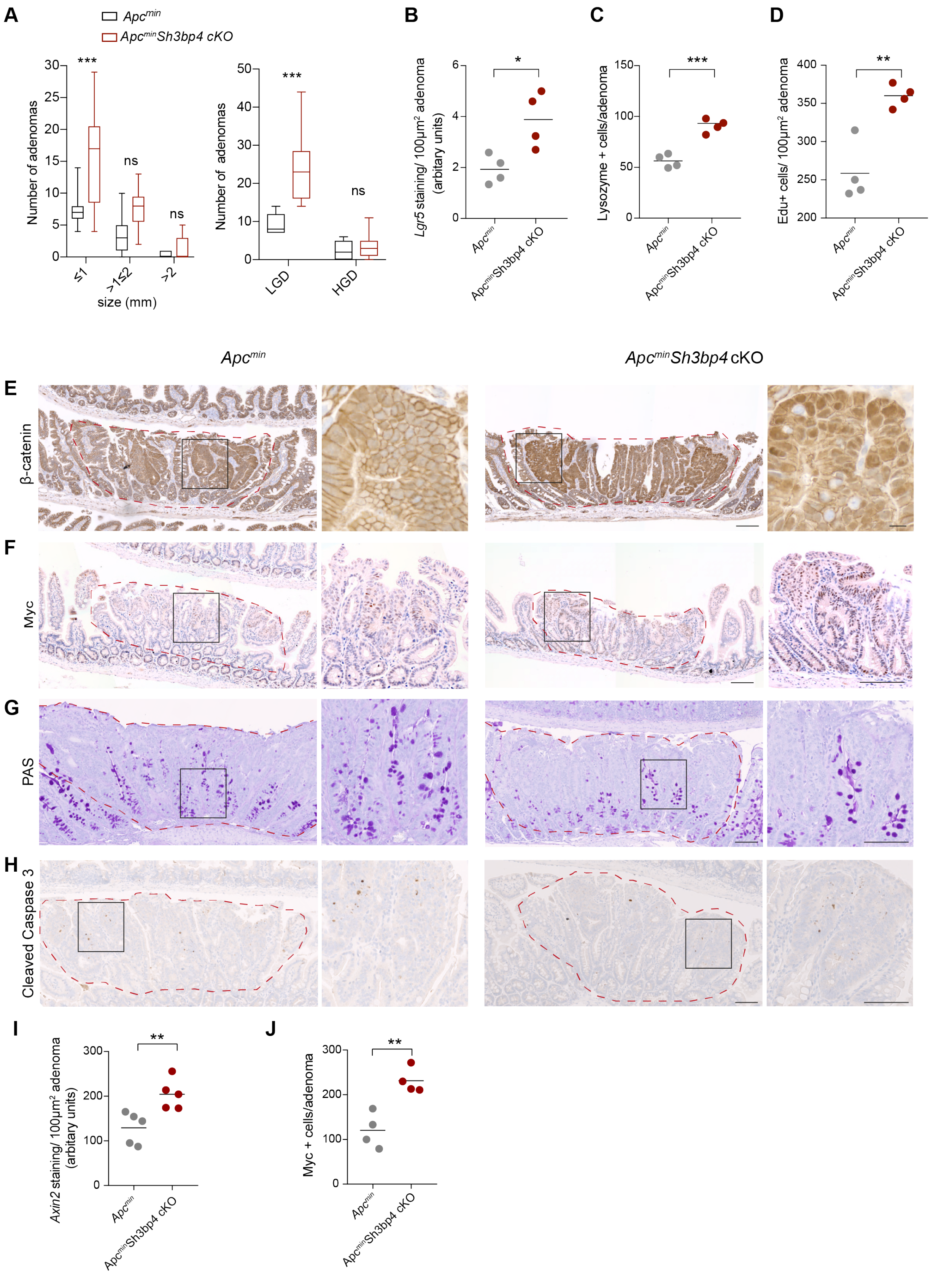

Figure S4. Increased Wnt/β-catenin signaling and reduced differentiation in *Apc^min^Sh3bp4* cKO adenomas, Related to Figure 3. (A) Distribution of adenomas categorised based on size (left) and grade of dysplasia (right). LGD, low grade dysplasia; HGD, high grade dysplasia. The adenoma classification as LGD and HGD are detailed in the Methods section. Data are represented as mean ±SD. ***P≤ 0.001, ns, non-significant. (B) Quantification of *Lgr5* staining per 100μm^2 adenoma in Figure 3D. (C) Quantification of Lysozyme$^+$ Paneth cells in Figure 3F. (D) Quantification of EdU+ proliferative cells in *Apc^{min}* and *Apc^{min}Sh3bp4* cKO adenomas in Figure 3G. Each dot represents the average of at least 5 adenomas (with similar size and grade of dysplasia) per animal. Black bar indicates the mean per group. n=4 animals/group. ***P≤ 0.001, **P≤ 0.01, *P≤0.05. (E-H) Immunohistochemistry staining of

β-catenin (E), Myc (F), PAS (G) and cleaved-caspase 3 (H) in Apc^{min} and Apc^{min}*Sh3bp4* cKO intestine. Increased nuclear β-catenin and Myc expression is observed in Apc^{min}*Sh3bp4* cKO adenomas compared to WT. Magnifications of the boxed area are shown. PAS staining shows reduced number of Goblet cells in adenomas with *Sh3bp4* deletion. Cleaved-caspase 3 staining show similar levels of apoptosis in Apc^{min} and Apc^{min}*Sh3bp4* cKO adenomas. Images are representative of at least 6 animals analyzed per group. Scale bar, 100µm. (I) Quantification of *Axin2* staining per 100□m^2 adenoma in Figure 3E. Each dot represents the average of at least 5 adenomas (with similar size and grade of dysplasia) per animal. Black bar indicates the mean per group. n=5 animals/group. (J) Quantification of Myc+ cells in Apc^{min} and Apc^{min}*Sh3bp4* cKO adenomas in Figure S4F. Each dot represents the average of at least 5 adenomas (with similar size and grade of dysplasia) per animal. Black bar indicates the mean per group. n=4 animals/group. $^{**}P \leq 0.01$.

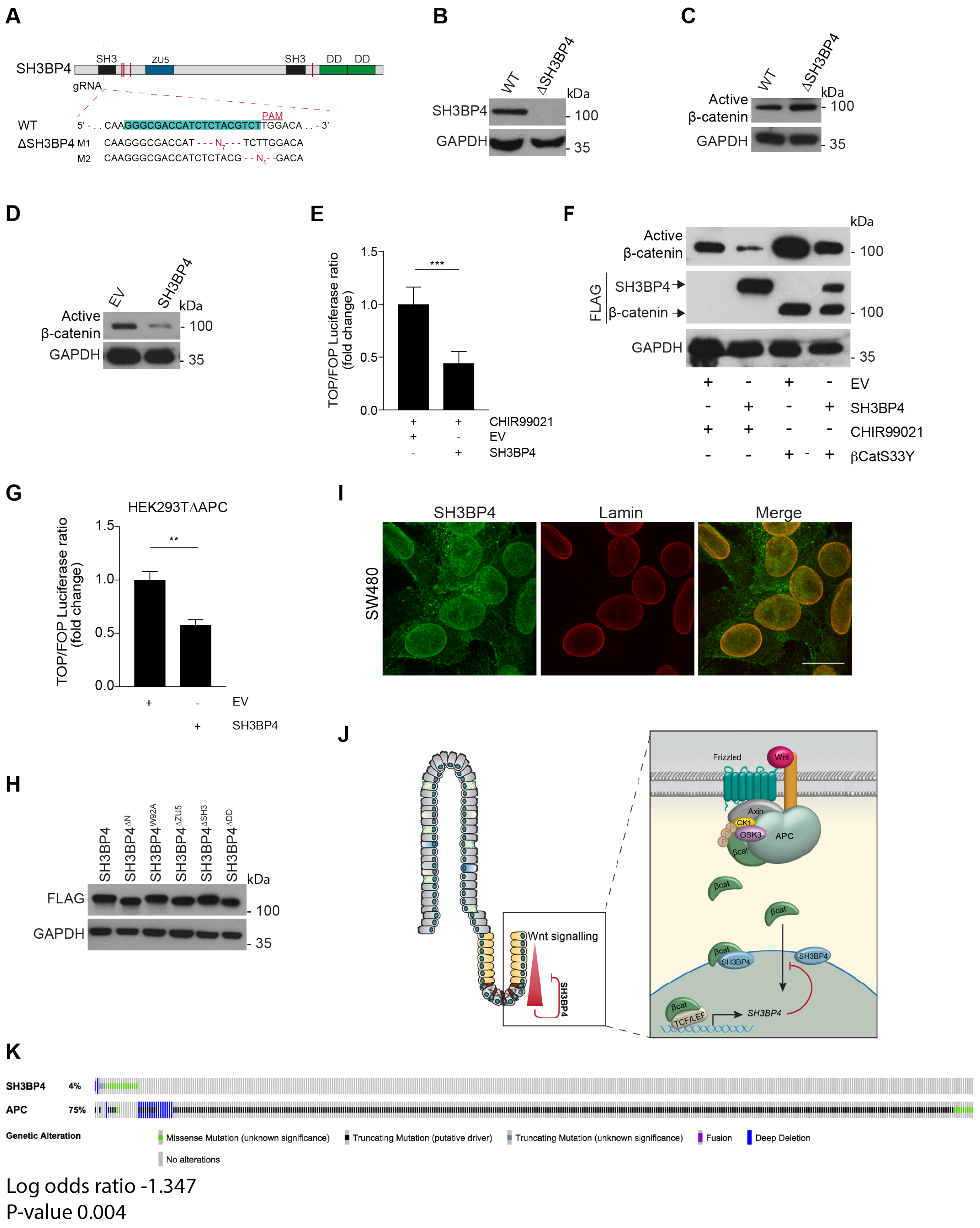

Figure S5. SH3BP4 negatively regulates Wnt signaling, Related to Figure 4. (A) Schematic representation of the CRISPR/Cas9 targeting strategy of *SH3BP4*. Genotyping of the selected ΔSH3BP4 mutant clone reveals homozygous frameshift deletions on both alleles, indicated as M1 and M2. A 7bp- and a 5bp deletion were retrieved from the ΔSH3BP4 clone, resulting in protein truncation at 110 and 89 amino acids respectively. (B) Immunoblot analysis showing protein loss in the ΔSH3BP4 mutant clone. (C) ΔSH3BP4 cells show increased levels of active-β-catenin in Wnt3A-induced HEK293T cells when compared to wild-type. (D) Ectopic expression of SH3BP4 in HEK293T cells shows reduced levels of active-β-catenin. (E) HEK293T cells overexpressing EV or SH3BP4 plasmids were treated with 2μM of GSK3 inhibitor CHIR99021 for 24 hours and relative TOPFlash reporter activity was measured. (F) Western blot shows that SH3BP4 reduces the active β-catenin levels induced by GSK3 inhibition (CHIR99021) and β-cateninS33Y expression. (G) SH3BP4 expression suppresses TOPFlash reporter activity induced by APC truncation (HEK293T ΔAPC) (Novellasdemunt et al., 2017). (H) Western blot analysis showing similar protein expression level among different FLAG-tagged SH3BP4 WT and mutant constructs. (I) Immunofluorescence of SH3BP4 (green) and the perinuclear marker Lamin (red) in SW480 cells. Scale bar, 100 μm. (J) Proposed model for the negative feedback

role of SH3BP4 in Wnt signaling. SH3BP4 is expressed at the Wnt-active stem cell compartment as a negative feedback mechanism to control Wnt/β-catenin signaling and maintain ISC homeostasis. SH3BP4 is present at the perinuclear membrane to restrict β-catenin nuclear translocation.

Table S1. Primer sequences, Related to STAR Methods.

REAGENT or RESOURCE	SOURCE	IDENTIFIER
Oligonucleotides		
SH3BP4, F: 5' gtcaaggtgtaggagagggagg 3', R: 5'ggcaaaggactcagaggaatg3'	This paper	N/A
SH3BP4$^{\Delta N}$ F: 5'gtggcggccgctcgaggccatgcccttgaactaccggaactcaacac3'; R: 5'ggcaaaggactcagaggaatg 3'	This paper	N/A
SH3BP4$^{\Delta DD}$ F:5' gtcaaggtgtaggagagggagg3' R:5' tccttgtagtctcgagcctccagcagcaccgaggtgctca3';	This paper	N/A
SH3BP4$^{\Delta SH3}$ F: 5'gtgtccagcctcaagctggtggtcggcagggcccgg3' R: 5'cctgccgaccaccagttgaggctggacaccgggcgg3'	This paper	N/A
SH3BP4$^{\Delta ZU5}$ F 5'cagacccaagccgtgctttttagcaaaagcacagtg3' R: 5'gcttttgctaaaaagcacggcttgggtctggcccca3'	This paper	N/A
SH3BP4^{W92A} F: 5' cacatctggcggtgaggcgtggtacgcacacaac 3', R: 5' gttgtgtgcgtaccacgcctcaccgccagatgtg	This paper	N/A
RT-qPCR *AXIN2*, F: 5' agtgtgaggtccacggaaac3', R: 5' cttcacactgcgatgcattt 3'	This paper	N/A
RT-qPCR *MYC* F: 5'gtcgtagtcgaggtcatag3', R: 5' tctccttgcagctgcttag3'	This paper	N/A
RT-qPCR *CCND1* F: 5'aaatatcccgacagggttcc3', R: 5'gtgttctctccgctgtaggc3'	This paper	N/A
RT-qPCR *Sh3bp4* F: 5'gtggcgtccgtcctagaaaa3', R: 5'gactcgtaggcgtccatctg3'	This paper	N/A
RT-qPCR *ACTIN* F: 5'gagcgcggctacagctt3', R: 5'tccttaatgtcacgcacgattt3'	This paper	N/A
SH3BP4 CRISPR/Cas9 gRNA: 5'gggcgaccatctctacgtct3'	This paper	N/A
