# Supplementary material for: SH3BP4 Regulates Intestinal Stem Cells and Tumorigenesis by Modulating β-Catenin Nuclear Localization
Source: Cell Rep. 2019 Feb 26;26(9):2266–2273.e4. doi: 10.1016/j.celrep.2019.01.110 (PMC6391711; doi:10.1016/j.celrep.2019.01.110)
Supplement: Document S2. Article plus Supplemental Information [file mmc2.pdf]

Cell Reports

Report

SH3BP4 Regulates Intestinal Stem Cells and Tumorigenesis by Modulating β-Catenin Nuclear Localization

Graphical Abstract

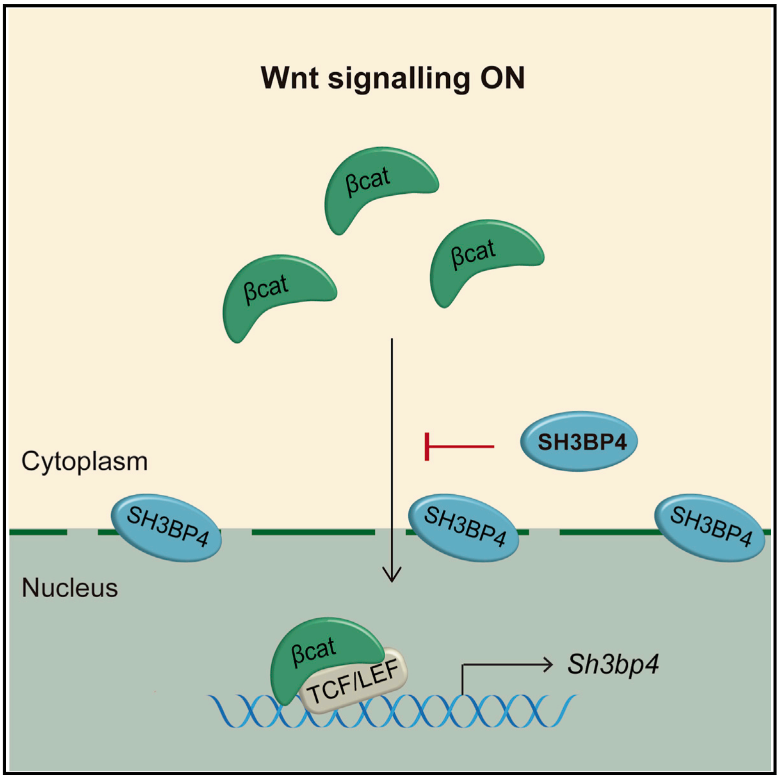

Highlights

- SH3BP4 is a Wnt inhibitor and is expressed in the intestinal crypt
- Deletion of Sh3bp4 increases stem cell numbers and accelerates tumor development
- SH3BP4 inhibits β-catenin nuclear localization at the perinuclear region

Authors

Pedro Antas, Laura Novellasdemunt, Anna Kucharska, ..., Emma Nye, Marco Novelli, Vivian S.W. Li

Correspondence

vivian.li@crick.ac.uk

In Brief

Antas et al. find that *SH3BP4* is transcriptionally activated by Wnt in the intestinal crypt to inhibit Wnt activity as a negative feedback mechanism. SH3BP4 restricts β-catenin nuclear localization at the perinuclear region. Deletion of Sh3bp4 increases stem cell numbers and accelerates intestinal tumor development in mice.

Antas et al., 2019, Cell Reports *26*, 2266–2273
February 26, 2019 © 2019 Francis Crick Institute.
https://doi.org/10.1016/j.celrep.2019.01.110

CellPress

OPEN ACCESS
CellPress

Cell Reports
Report

SH3BP4 Regulates Intestinal Stem Cells and Tumorigenesis by Modulating β-Catenin Nuclear Localization

Pedro Antas,[1] Laura Novellasdemunt,[1] Anna Kucharska,[1] Isobel Massie,[1] Joana Carvalho,[1] Dahmane Oukrif,[2] Emma Nye,[1] Marco Novelli,[2] and Vivian S.W. Li[1,3,*]
[1]The Francis Crick Institute, 1 Midland Road, London NW1 1AT, UK
[2]Histopathology Department, University College London Hospitals NHS Foundation Trust, London, UK
[3]Lead Contact
*Correspondence: vivian.li@crick.ac.uk
https://doi.org/10.1016/j.celrep.2019.01.110

SUMMARY

Wnt signals at the base of mammalian crypts play a pivotal role in intestinal stem cell (ISC) homeostasis, whereas aberrant Wnt activation causes colon cancer. Precise control of Wnt signal strength is governed by a number of negative inhibitory mechanisms acting at distinct levels of the cascade. Here, we identify the Wnt negative regulatory role of Sh3bp4 in the intestinal crypt. We show that the loss of *Sh3bp4* increases ISC and Paneth cell numbers in murine intestine and accelerates adenoma development in *Apc^min^* mice. Mechanistically, human SH3BP4 inhibits Wnt signaling downstream of β-catenin phosphorylation and ubiquitination. This Wnt inhibitory role is dependent on the ZU5 domain of SH3BP4. We further demonstrate that SH3BP4 is expressed at the perinuclear region to restrict nuclear localization of β-catenin. Our data uncover the tumor-suppressive role of SH3BP4 that functions as a negative feedback regulator of Wnt signaling through modulating β-catenin's subcellular localization.

INTRODUCTION

In mammalian intestine, Wnt ligands and the agonist R-spondin are secreted at the crypt bottom to generate a Wnt gradient radiating from stem cells to the trans-amplifying zone at the crypt-villus junction. On the other hand, a number of negative regulators acting at distinct levels of the cascade are present to restrict Wnt signal at the crypt bottom. These include the previously reported Wnt inhibitors, such as AXIN2 (Jho et al., 2002), RNF43 (Koo et al., 2012), and APCDD1 (Shimomura et al., 2010). They are direct Wnt targets expressing in the Wnt-active intestinal stem cell (ISC) region. These inhibitors negatively regulate the pathway and are considered as tumor suppressors in various human cancers (Giannakis et al., 2014; Cancer Genome Atlas Network, 2012; Yan et al., 2017). These findings suggest that a class of stem-cell-expressed Wnt target genes function as negative-feedback regulators in the crypt to fine-tune Wnt/β-catenin signaling for ISC maintenance. Here, we describe the discovery of SH3 domain-binding protein 4 (SH3BP4) that is expressed in the Wnt-active intestinal crypt and negatively regulates Wnt/β-catenin signaling.

SH3BP4 has been previously suggested as a potential tumor suppressor gene in multiple human cancers, including breast, renal, and non-small-cell lung cancers with a high frequency of deletion (Kim et al., 2012). SH3BP4 plays a regulatory role in a number of signaling pathways, including clathrin-mediated internalization of the transferrin receptor (TfR), fibroblast growth factor receptor (FGFR) trafficking, and amino acid-Rag GTPase-mechanistic target of rapamycin 1 (mTORC1) signaling (Francavilla et al., 2013; Kim et al., 2012; Tosoni et al., 2005). The role of SH3BP4 in Wnt/β-catenin signaling and ISC homeostasis has never been explored. In this study, we examine the role of SH3BP4 in Wnt signal regulation in the context of intestine. The loss of Sh3bp4 increases ISC numbers and augments tumorigenesis with increased Wnt activity. We show that SH3BP4 negatively regulates Wnt signaling by modulating nuclear localization of β-catenin. Our findings provide the mechanistic insight into the role of Sh3bp4 in ISCs and cancer by regulating Wnt/β-catenin signaling.

RESULTS

Expression of *Sh3bp4* in the Wnt-Active Intestinal Crypt

We first characterized the expression of *Sh3bp4* in the intestine. qRT-PCR on mouse intestinal epithelium showed that *Sh3bp4* was enriched in the crypt fraction similar to other ISC markers, namely, *Lgr5* and *Olfm4* (Figure S1A). RNAscope *in situ* hybridization (ISH) further showed the crypt expression of *Sh3bp4* in both small intestine and colon (Figures 1A and 1B). RNAscope co-staining analysis further revealed that *Sh3bp4* was co-localized with the ISC marker *Lgr5* (Figure 1C), which was confirmed by qRT-PCR of sorted *Lgr5*-GFP cells (Figure 1D).

Because *Sh3bp4* is expressed in the Wnt-active crypt bottom, we asked if *Sh3bp4* is regulated by Wnt signaling. RNAscope analysis of *Apc^min^* intestine showed upregulation of *Sh3bp4* in adenomas with aberrant Wnt activation, suggesting that *Sh3bp4* expression is modulated by Wnt signaling (Figure 1E). Consistently, expression of *Sh3bp4* was also upregulated in *Apc* mutant organoids (ΔAPC) generated by CRISPR targeting (Figure 1F) (Novellasdemunt et al., 2017), as well as in HEK293T cells upon Wnt3A stimulation (Figures S1B and S1C).

 © 2019 Francis Crick Institute.
This is an open access article under the CC BY license (http://creativecommons.org/licenses/by/4.0/).

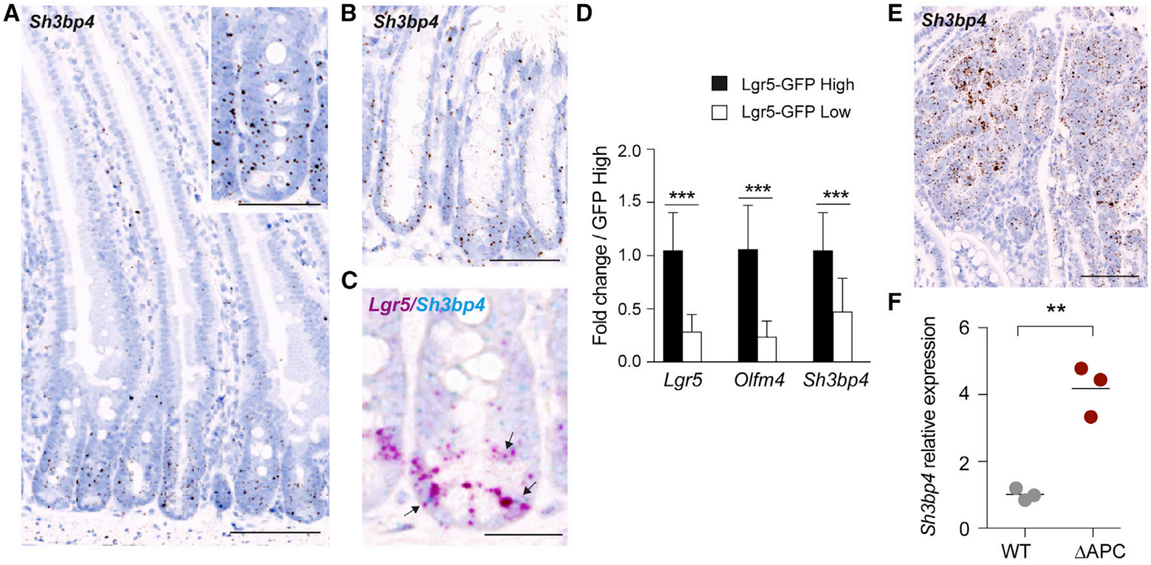

Figure 1. *Sh3bp4* Is a Stem Cell-Expressed Wnt Target Gene

(A and B) Representative image of RNAscope ISH showing *Sh3bp4* gene expression in small intestine (A) and colon (B).

(C) Representative RNAscope image showing co-localization of *Lgr5* (red) and *Sh3bp4* (blue) gene expression (indicated by black arrows).

(D) qRT-PCR showing fold change of stem-cell genes (*Lgr5* and *Olfm4)* and *Sh3bp4* in sorted Lgr5-GFP crypt cells from 6 biological replicates.

(E) Representative image of RNAscope ISH showing increased expression of *Sh3bp4* in Apc^{min} adenomas.

(F) qPCR showing increased expression of *Sh3bp4* in *Apc*-mutated organoids compared to WT organoids (n = 3).

Scale bars, 100 μm; insets, 50 μm. Data are represented as mean ± SD. ***p ≤ 0.001, **p ≤ 0.01. See also Figure S1.

The Wnt-induced expression of SH3BP4 can be suppressed upon Wnt inhibitor LF3 treatment (Figure S1C), suggesting that SH3BP4 is Wnt transcriptional target. In addition, the upregulated expression of SH3BP4 was also observed in human colorectal cancer (CRC) tissues and the Wnt-activated CRC cell lines (Figures S1D and S1E). Transcriptomic analysis of human CRC patients further confirmed the increased expression of *SH3BP4* in tumor samples (Figure S1F) (Cancer Genome Atlas Network, 2012).

To demonstrate *SH3BP4* is transcriptionally regulated by Wnt, we analyzed the TCF7L2/TCF4 chromatin immunoprecipitation sequencing (ChIP-seq) data generated from two different human CRC cell lines, namely, Ls174T and HCT116 (ENCODE Project Consortium, 2012; Hatzis et al., 2008). Multiple TCF4-binding sites were identified upstream and throughout the gene locus of *SH3BP4* and were co-localized with the active enhancer regions (H3K27Ac), suggesting that they are active TCF4-binding motifs for gene transcription (Figure S1G). Together, these data suggest that *SH3BP4* is expressed in the Wnt-active intestinal crypt and is transcriptionally activated by Wnt signaling.

Loss of *Sh3bp4* Increases the Number of ISCs and Paneth Cells

To investigate the functional role of SH3BP4 in intestinal homeostasis, we crossed $Sh3bp4^{fl/fl}$ mice to $Villin^{CreERT2}$ mice to generate intestine-specific conditional knockout $Villin^{CreERT2}Sh3bp4^{fl/fl}$ (*Sh3bp4* cKO) animals (Figure S2A). RNAScope analysis confirmed efficient loss of *Sh3bp4* upon tamoxifen induction (Figure S2B). *Sh3bp4 cKO* intestine, 25 days post-induction, showed increased expression of the stem cell marker and Wnt target *Lgr5* when compared with $Sh3bp4^{fl/fl}$ control littermates (hereafter named as wild-type [WT]) (Figures 2A–2D). The increase in ISC number was further confirmed by another stem cell marker, *Olfm4* (Figures 2E–2H and 2M). Of note, the increase in ISC number was consistently observed 3 months after deletion of *Sh3bp4* (Figures S2C and S2D). Because Paneth cells constitute the niche for ISC maintenance (Sato et al., 2011), we asked if the increase in ISC population was accompanied by an increase in Paneth cell number. Indeed, increased Paneth cell number was observed in *Sh3bp4* cKO intestine, as revealed by lysozyme staining, suggesting that the loss of *Sh3bp4* results in an expansion of ISCs and their niche (Figures 2I–2L and 2N). We further assessed the clonogenicity of organoids derived from WT and *Sh3bp4* cKO intestinal crypts, which can be used as a functional readout of stem cell numbers (Sato et al., 2009). *Sh3bp4*-depleted organoids formed nearly two-fold more clones than the WT ones, indicating that there are significantly more stem cells in the mutant intestine (Figure 2O).

Besides the increase in the number of ISCs and Paneth cells, no other gross morphological changes were observed in *Sh3bp4*-depleted intestine (Figure S2E). The number of goblet cells and enteroendocrine cells were comparable between mutant and WT intestine, suggesting that differentiation is not affected upon *Sh3bp4* deletion (Figures S2F and S2G). Interestingly, crypt proliferation was not altered despite the increase in ISC numbers (Figures S2H–S2K). On the other hand, a significant increase in the number of individual crypts per circumference was observed in *Sh3bp4* cKO intestine (Figures S2L–S2N). Previous studies have shown that crypt fission is a mechanism to cope with accelerated mutant clonal expansion and epithelial

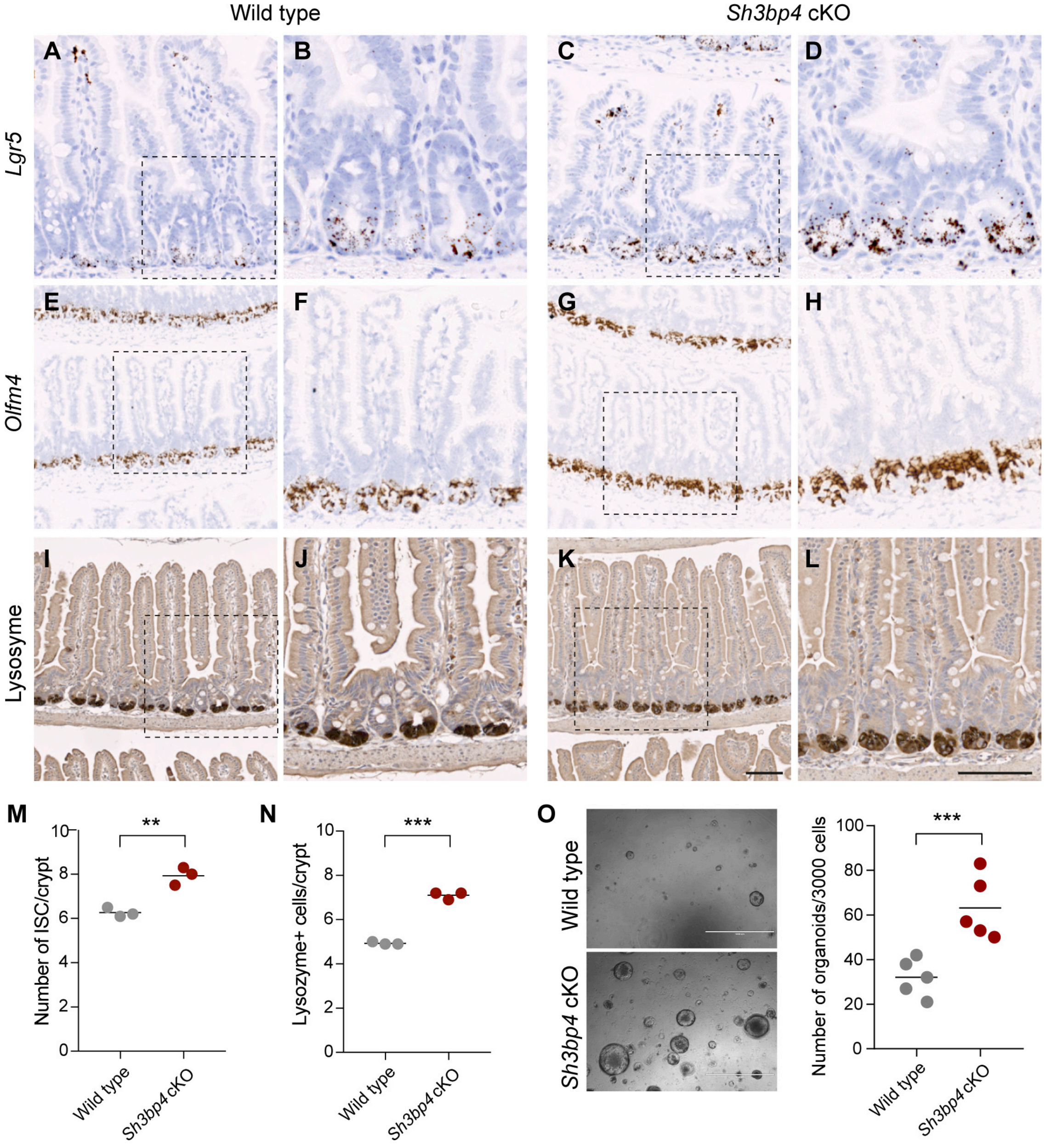

Figure 2. Loss of *Sh3bp4* Increases the Number of ISCs and Paneth Cells

(A–H) Histology analysis of WT (A, B, E, F, I, and J) and *Sh3bp4* cKO (C, D, G, H, K, and L) intestine. Representative images of RNAscope ISH of the stem cell markers *Lgr5* (A–D) and *Olfm4* (E–H).

(I–L) Immunohistochemistry of lysozyme representing Paneth cells. Images are representative of at least 6 animals analyzed per group. Scale bar, 100 μm. (B, D, F, H, J, and L) High-magnification images of boxed area in (A, C, E, G, I, and K), respectively.

(M) Quantitation of number of *Olfm4*$^+$ ISCs per crypt.

(N) Quantitation of number of lysozyme$^+$ Paneth cells. Each dot represents the average number of cells per crypt per animal (determined from at least 30 crypts per animal). Black bar shows the mean per group. n = 3 per group.

(O) Microscopy images of the organoids derived from WT and *Sh3bp4* cKO animals. Number of organoids formed per 3,000 single cells.

Each dot represents the average of 3 triplicates per animal. Black bar indicates the mean per group. n = 5 animals/group. ***p $\leq$ 0.001, **p $\leq$ 0.01. See also Figures S2 and S3.

colonization (Nicholson et al., 2018; Snippert et al., 2014). Our data suggest that the loss of *Sh3bp4* leads to an expansion of ISC compartment, which then causes accelerated crypt fission.

SH3BP4 has previously been reported as a negative regulator of mTOR1 signaling (Kim et al., 2012). We asked if the increase in the number of ISCs upon *Sh3bp4* loss is caused by upregulated

mTOR signaling. *Sh3bp4* cKO and WT animals were treated with vehicle or mTOR inhibitor rapamycin for 30 days, and intestinal tissues were collected for histology analysis (Figure S3A). Inhibition of mTOR signaling was confirmed by the loss of phosphorylation of the mTOR effector RPS6 (pS6) in both *Sh3bp4* cKO and WT intestine (Figure S3B). Interestingly, suppression of mTOR signaling did not rescue the ISC expansion phenotype in *Sh3bp4* cKO intestine, suggesting that the increase in ISC numbers upon *Sh3bp4* loss is independent of mTOR signaling (Figures S3C–S3E).

Sh3bp4 Deletion Augments Tumorigenesis in *Apc*^{min} Animals by Enhancing Wnt Activation and Increasing the Number of ISCs and Paneth Cells

Next, we examined if SH3BP4 plays a role in intestinal tumorigenesis. *Sh3bp4* cKO mice were crossed to the intestinal tumor model *Apc*^{min} mice (*Apc*^{min}*Sh3bp4* cKO). Tamoxifen was administered to the *Apc*^{min} and *Apc*^{min}*Sh3bp4* cKO animals (n = 10/group) at 6 weeks old, and mice were sacrificed at predetermined humane endpoints. Although *Apc*^{min} mice lived for 5–6 months (165 days on average), *Apc*^{min}*Sh3bp4* cKO mice started showing signs of sickness much earlier by age 3–5 months (125 days on average) (Figure 3A). *Apc*^{min}*Sh3bp4* cKO mice (n = 13) exhibited more than 2-fold increase in total adenoma numbers in small intestine compared to control *Apc*^{min} littermates (n = 7) (Figures 3B and 3C). Most adenomas were low-grade dysplasias (LGD), whereas *Apc*^{min}*Sh3bp4* cKO mice had a moderate increase in the number of adenomas with high-grade dysplasias (HGD) although not significant (Figure S4A).

Because the loss of *Sh3bp4* alone caused ISC expansion, we further analyzed the stem cell marker *Lgr5* expression in *Apc*^{min} and *Apc*^{min}*Sh3bp4* cKO adenomas. Consistent with the *Sh3bp4* cKO phenotype, a significant increase in *Lgr5*-expressing stem cells was observed in the *Apc*^{min}*Sh3bp4* cKO adenomas (Figures 3D and S4B). We further asked if Wnt signaling is hyperactivated upon *Sh3bp4* deletion. Increased nuclear localization of β-catenin was observed in most *Apc*^{min}*Sh3bp4* cKO adenomas (Figure S4E). This was accompanied by upregulated expression of Wnt targets *Axin2* and Myc in *Apc*^{min}*Sh3bp4* cKO adenomas (Figures 3E, S4F, S4I, and S4J), suggesting that Wnt signaling is hyperactivated upon *Sh3bp4* deletion. The number of lysozyme+ Paneth cells was also significantly increased in *Apc*^{min}*Sh3bp4* cKO adenomas compared to the control *Apc*^{min} littermates (Figures 3F and S4C). On the other hand, goblet cell number was reduced, suggesting that differentiation is suppressed in *Sh3bp4*-depleted tumors (Figure S4G). In addition, *Apc*^{min}*Sh3bp4* cKO adenomas further displayed increased proliferation, as indicated by 5-ethynyl-2′-deoxyuridine (EdU)+ cells, whereas apoptosis was not affected (Figures 3G, S4D, and S4H). Together, our findings indicate that the loss of *Sh3bp4* in an *Apc*^{min} background promotes intestinal tumorigenesis by enhancing Wnt activation and expanding ISC and Paneth cell populations.

SH3BP4 Inhibits Wnt Signaling by Modulating Nuclear Localization of β-Catenin by the ZU5-Domain

To investigate how SH3BP4 regulates Wnt/β-catenin signaling, we first generated SH3BP4 knockout (ΔSH3BP4) in HEK293T cells by using the CRISPR/Cas9 system (Figures S5A and S5B). The loss of SH3BP4 resulted in a ~2.5-fold increase in Wnt3A-induced TOPFlash reporter transcriptional activity (Figure 4A), as well as an increase in active β-catenin protein levels (Figure S5C). Consistently, significantly upregulated expression of Wnt target genes *AXIN2*, *CCND1*, and *MYC* was detected in ΔSH3BP4 cells compared to WT (Figure 4B). Next, we performed ectopic expression of SH3BP4 in HEK293T cells and found significant suppression of Wnt3A-induced TOPFlash activity and the active β-catenin protein level (Figures 4C and S5D). The results indicate that SH3BP4 negatively regulates Wnt/β-catenin signaling. To understand how SH3BP4 regulates the Wnt pathway, we examined the inhibitory effect of SH3BP4 on the signaling cascade at different subcellular levels by using various Wnt activation models. In brief, Wnt activation was achieved by (1) expressing a mutant form of the LRP6 receptor lacking the extracellular domain (ΔN-LRP6) (Liu et al., 2003), (2) expressing a constitutively active form of β-catenin (βCatS33Y), (3) pharmacological inhibition of GSK3 activity by using CHIR99021, or (4) inhibition of β-catenin ubiquitination upon *APC* truncating mutation (HEK293TΔAPC) (Novellasdemunt et al., 2017). Interestingly, ectopically expressed SH3BP4 was able to suppress Wnt activation mediated by a mutant receptor (ΔN-LRP6) (Figure 4D), inhibition of β-catenin phosphorylation (βCatS33Y and CHIR99021) (Figures 4E, S5E, and S5F), and ubiquitination (HEK293TΔAPC) (Figure S5G). The data support the idea that SH3BP4 inhibits Wnt signaling downstream of phosphorylation and ubiquitination of β-catenin.

To define the region of SH3BP4 that is important for Wnt signal inhibition, we generated different SH3BP4 truncating mutations and a point mutant (SH3BP4^{W92A}) destroying the first SH3 domain-specific interaction (Figure 4F) (Erpel et al., 1995; Tosoni et al., 2005). Comparable protein expression levels were observed across all mutant constructs (Figure S5H). We then ectopically expressed WT or mutant SH3BP4 constructs in the SH3BP4-deficient cells (ΔSH3BP4) and assessed the Wnt3A-induced TOPFlash reporter activity. As expected, expression of WT SH3BP4 readily suppressed the Wnt activation caused by endogenous SH3BP4 depletion (Figure 4F). Similar to the WT protein, most of the SH3BP4 mutants were also able to repress Wnt activation except the ZU5-lacking mutant (SH3BP4$^{\Delta ZU5}$), suggesting that the Wnt inhibitory role of SH3BP4 is dependent on the ZU5 domain (Figure 4F). This result was further confirmed in the HEK293T ΔAPC Wnt activating model, where SH3BP4$^{\Delta ZU5}$ failed to inhibit Wnt signaling induced by APC truncation (Figure 4G). Together, we conclude that SH3BP4 inhibits Wnt signaling downstream of β-catenin phosphorylation and ubiquitination and is dependent on its ZU5 domain.

Previous studies have shown that the SH3BP4 protein is localized to the plasma membrane, perinuclear region, and clathrin-coated vesicles (Kim and Kim, 2013; Kim et al., 2012; Tosoni et al., 2005). In concordance with the previous studies, we confirmed that WT SH3BP4 was predominantly expressed at the perinuclear region (Figure 4H). We further confirmed the perinuclear expression of SH3BP4 endogenously in the Wnt-activated SW480 CRC cells (Figure S5I). In contrast, the SH3BP4$^{\Delta ZU5}$ mutant was localized to the plasma membrane and cytoplasm instead, suggesting that the ZU5 domain is required for perinuclear localization of the SH3BP4 protein (Figure 4H). Interestingly,

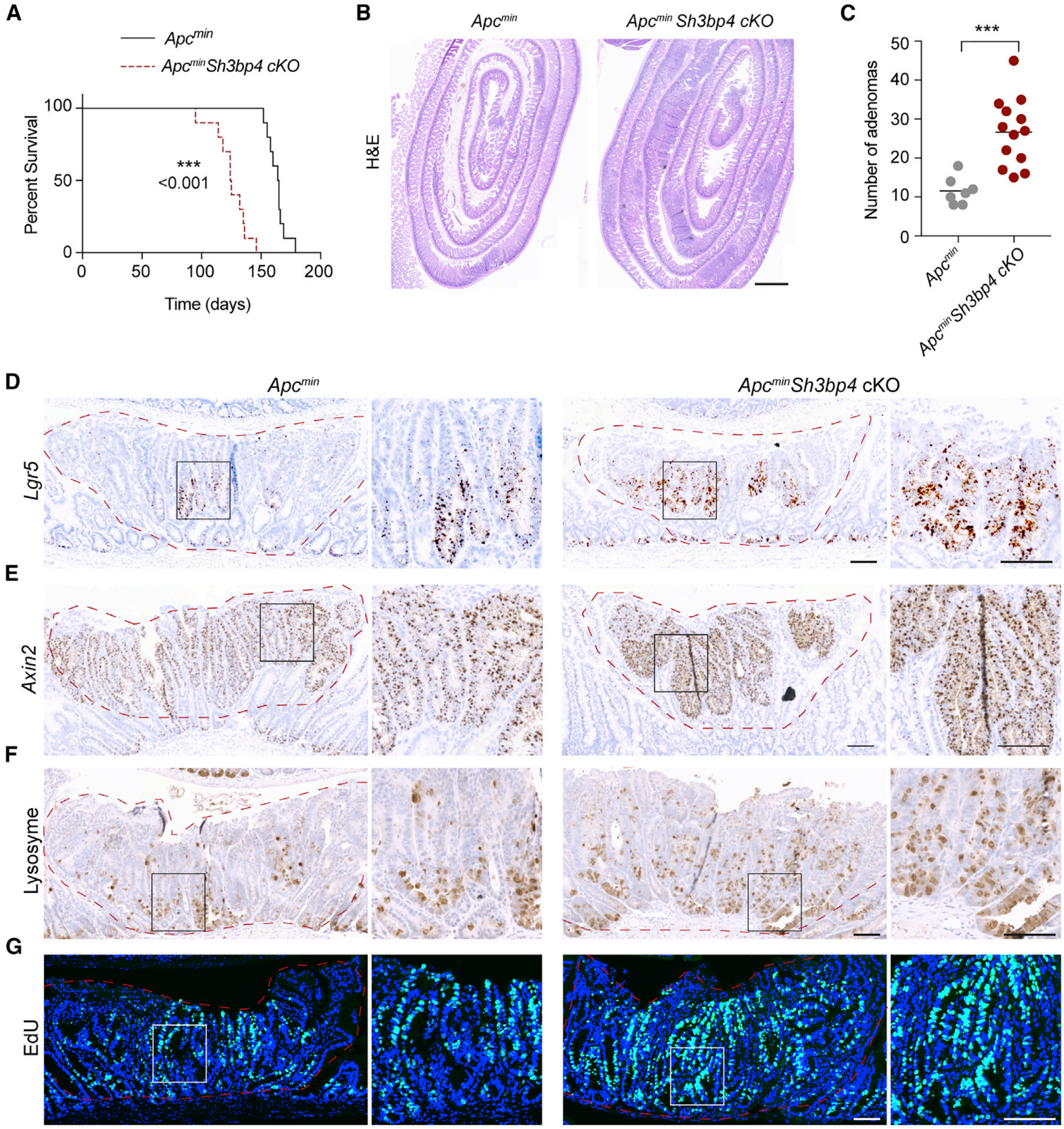

Figure 3. *Sh3bp4* Deletion Augments Tumorigenesis in *Apc*^{min} Animals by Enhancing Wnt Signaling and ISC Numbers

(A) Kaplan-Meier survival analysis of *Apc*^{min} and *Apc*^{min}*Sh3bp4* cKO mice. Loss of SH3BP4 was induced 6 weeks after birth (n = 10).

(B) Representative H&E-stained sections of small intestine from *Apc*^{min} (left) and *Apc*^{min}*Sh3bp4* cKO (right) mice. Scale bar, 1 mm.

(C) Total number of adenomas in the intestine 2 months after induced SH3BP4 loss. Each dot represents the number of adenomas present per animal. *Apc*^{min} (n = 7), *Apc*^{min}*Sh3bp4* cKO (n = 13). Mean was indicated by black bars.

(D and E) Representative images of RNAscope ISH of stem cell and Wnt target genes *Lgr5* (D) and *Axin2* (E) in *Apc*^{min} (left) and *Apc*^{min}*Sh3bp4* cKO (right) mice. Magnifications of the boxed adenomas region are shown.

(F and G) Immunohistochemistry staining of lysozyme (F) and EdU (G) in the indicated tissues. Images are representative of at least 6 animals analyzed per group. Scale bars, 100 μm. See also Figure S4.

ectopic expression of WT SH3BP4 in ΔAPC cells suppressed the nuclear β-catenin level and enhanced perinuclear accumulation of β-catenin that co-localized with SH3BP4 (Figure 4H). On the other hand, the nuclear β-catenin level was not affected in SH3BP4$^{\Delta ZU5}$-expressing cells (Figure 4I). To validate the findings, we further examined the β-catenin protein level in different subcellular fractions by using western blot analysis. Consistent with the immunofluorescent data, expression of WT SH3BP4 (but not SH3BP4$^{\Delta ZU5}$) significantly suppressed the β-catenin level in the nuclear fraction (Figures 4J and 4K). Our results indicate that SH3BP4 is expressed at the perinuclear region to control nuclear translocation of β-catenin. Deletion of ZU5 fails to

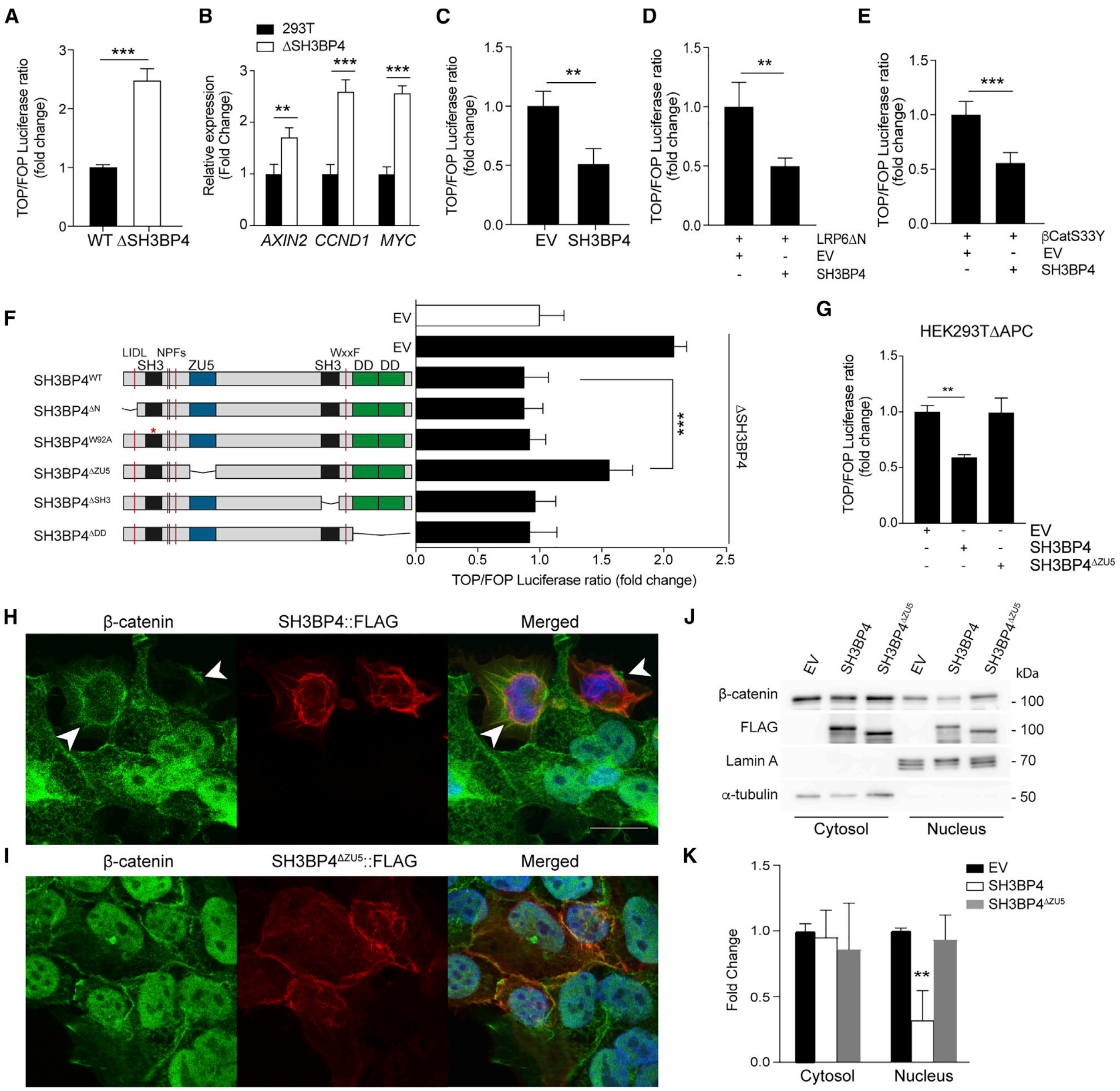

Figure 4. SH3BP4 Inhibits Wnt Signaling by Modulating Nuclear Translocation of β-Catenin by Its ZU5 Domain

(A) Relative Wnt3a-induced TOPFlash reporter activity in HEK293T wild-type and ΔSH3BP4 cells.

(B) qRT-PCR of Wnt target genes *AXIN2*, *CCND1*, and *MYC* in the indicated cells. Expression data are presented as fold induction normalized to β-actin.

(C–E) TOPFlash reporter activity upon ectopic expression of the indicated plasmids. Wnt signal is induced by Wnt3A treatment (C), expression of ΔN-LRP6 (D), or β-CatS33Y (E).

(F) Relative Wnt3a-induced TOPFlash reporter activity in WT (white bar) or ΔSH3BP4 (black bars) cells. Expression of WT or mutant SH3BP4 plasmids indicated on the left. EV, empty vector.

(G) TOPFlash reporter activity upon ectopic expression of the indicated plasmids in HEK293TΔAPC cells. Data represent average ± SD of at least three independent experiments.

(H and I) Immunofluorescence of β-catenin (green), FLAG-SH3BP4 (red), and 4′,6-diamidino-2-phenylindole (DAPI) (blue) in HEK293TΔAPC cells. Expression of WT SH3BP4 alters localization of β-catenin from nuclear to perinuclear membrane (arrow head) (H), whereas SH3BP4$^{\Delta ZU5}$ does not affect β-catenin nuclear localization (I). Scale bar, 100 μm.

(J) Western blot analysis of cytoplasmic-nuclear fractionation of HEK293TΔAPC cells expressing empty-vector (EV), SH3BP4, or SH3BP4$^{\Delta ZU5}$ by using indicated antibodies.

(K) Quantitation of the β-catenin protein levels in (J). Fold change of β-catenin level was relative to tubulin (cytosol) or lamin A (nucleus) (n = 3).

Data represent mean ± SD. ***$p \leq 0.001$, **$p \leq 0.01$. See also Figure S5.

localize SH3BP4 to the perinuclear region, thereby abrogating its ability to regulate β-catenin nuclear shuttling.

DISCUSSION

In this study, we uncover the negative feedback role of SH3BP4 in Wnt signaling for intestinal homeostasis and tumorigenesis (Figure S5J). *Sh3bp4* is expressed in the intestinal crypt under Wnt signal regulation. We show that SH3BP4 negatively regulates Wnt signaling at the perinuclear region by restricting β-catenin nuclear translocation by its ZU5 domain. Deletion of *Sh3bp4* increases the number of ISCs and Paneth cells, which is independent of mTOR signaling. Loss of *Sh3bp4* exacerbates the *Apc^min^* tumor phenotype through hyperactivation of Wnt signaling, suggesting its tumor suppressive role in colorectal cancer. Our findings highlight the crucial role of the negative feedback mechanism in both stem cell and cancer.

Previous studies have identified several Wnt inhibitors, such as AXIN2 and RNF43, that are expressed in the stem cell region to repress Wnt signaling at the cytoplasmic destruction complex and receptor levels, respectively (Jho et al., 2002; Koo et al., 2012). Our current study unveils another crypt-expressed Wnt inhibitor, SH3BP4, which contributes to the negative feedback loop at the nuclear level. Regulation of the Wnt signal cascade has been extensively characterized in the past, yet it remains elusive how β-catenin nuclear translocation is controlled. Given that β-catenin degradation is restricted to the cytoplasm, the regulation of β-catenin nuclear export is, thus, likely to be an important additional mechanism for Wnt signal regulation. Several studies have previously reported the APC-mediated nuclear export of β-catenin (Henderson, 2000; Rosin-Arbesfeld et al., 2000). Very recently, RAPGEF5 has further been reported to facilitate nuclear transport of β-catenin by activating the nuclear GTPase (Griffin et al., 2018). Our current findings define the ZU5-dependent role of SH3BP4 in negatively regulating Wnt signaling by modulating nuclear transportation of β-catenin. How SH3BP4 regulates β-catenin transportation at the perinuclear region remains to be determined. Interestingly, SH3BP4 has also been identified as a Rag GTPase-binding protein (Kim et al., 2012). Further studies will be needed to address if the role of SH3BP4 in regulating β-catenin nuclear transport is dependent on RAPGEF5 and/or APC.

Our current data show that ZU5 is the critical domain for modulating β-catenin nuclear localization. The ZU5 domain has been found in a wide range of proteins and has been implicated in protein-protein interactions. The ZU5 domain often exists together with a C-terminal death domain in proteins related to extracellular signal transduction, such as netrins (Reed et al., 2004; Wang et al., 2009), and in scaffold proteins, such as ankyrins (Ipsaro et al., 2009). The N-terminal SH3_1 domain of SH3BP4 has been previously reported to interact with Rag GTPases for mTOR inhibition (Kim et al., 2012), whereas its function is dispensable for Wnt signaling suppression. Together, the data corroborate the notion that the regulatory role of SH3BP4 in the Wnt pathway is independent of mTOR signaling. Whether the ZU5 domain might interact with any of the previously reported β-catenin nuclear transport proteins, such as APC and RAPGEF5, deserves investigation.

SH3BP4 is upregulated in many CRCs as a consequence of hyperactivation of Wnt signaling. Our current data suggest that SH3BP4 is able to inhibit Wnt signaling activated by APC or β-catenin mutations, which raises questions about why SH3BP4 fails to suppress Wnt activity in CRC cells. One possible explanation is that cancer cells may be addicted to the aberrant Wnt activation induced by oncogenic mutations, such as *APC*, which outcompetes the negative feedback signals to maintain the pathological Wnt activity. This perhaps is not so surprising considering that the other well-known Wnt inhibitor AXIN2 is also highly expressed in CRC cells. It is possible that such a negative feedback mechanism plays a gate-keeping role for fine-tuning the Wnt signal under normal homeostasis, whereas the role of these Wnt inhibitors might be less significant in cancer cells when Wnt activity passes beyond the pathological threshold. An alternative explanation is that the SH3BP4-mediated β-catenin nuclear shuttling mechanism might be inactivated in CRCs. In fact, inactivating mutations or deletion of these Wnt inhibitors (e.g., *AXIN2* and *RNF43*) have been previously identified in human CRCs (Giannakis et al., 2014; Cancer Genome Atlas Network, 2012; Yan et al., 2017), indicating their tumor suppressive roles in cancer. Interestingly, deletion and mutations of *SH3BP4* have also been reported in various cancers, including CRCs (Kim et al., 2012; Cancer Genome Atlas Network, 2012), and are mutually exclusive with *APC* mutations (Figure S5K). It is conceivable that SH3BP4 inactivation may contribute to an alternative Wnt activating mechanism in certain CRC subtypes, which could offer a new therapeutic strategy for targeting Wnt signaling in cancer.

STAR★METHODS

Detailed methods are provided in the online version of this paper and include the following:

- KEY RESOURCES TABLE
- CONTACT FOR REAGENT AND RESOURCE SHARING
- EXPERIMENTAL MODEL AND SUBJECT DETAILS
  - Animals
- METHOD DETAILS
  - Cell culture, transfection and TOPFlash assay
  - Crypts/Villi fractionation and organoid culture
  - Plasmids and reagents
  - CRISPR/Cas9 genome engineering
  - Antibodies and western blot analysis
  - Real-time quantitative RT-PCR
  - Immunofluorescence
  - Histology and Immunohistochemistry
  - RNAScope *in situ* hybridization
- QUANTIFICATION AND STATISTICAL ANALYSIS

SUPPLEMENTAL INFORMATION

Supplemental Information can be found with this article online at https://doi.org/10.1016/j.celrep.2019.01.110.

ACKNOWLEDGMENTS

We thank the Biological Research Facilities, Experimental Histopathology, and Flow Cytometry at the Francis Crick Institute for providing reagents and

technical assistance. This work was supported by the Francis Crick Institute, which receives its core funding from Cancer Research UK (FC001105), the UK Medical Research Council (FC001105), and the Wellcome Trust (FC001105). Work in the V.S.W.L. laboratory was also supported by the European Union's Horizon 2020 Research and Innovation programme (668294).

AUTHOR CONTRIBUTIONS

P.A. and V.S.W.L. designed the experiments and analyzed the data. P.A., L.N., A.K., I.M., J.C., D.O., E.N., and M.N. conducted the experiments. P.A. and V.S.W.L. wrote the paper.

DECLARATION OF INTERESTS

The authors declare no competing interests.

Received: August 24, 2018
Revised: January 9, 2019
Accepted: January 29, 2019
Published: February 26, 2019

REFERENCES

Barker, N., van Es, J.H., Kuipers, J., Kujala, P., van den Born, M., Cozijnsen, M., Haegebarth, A., Korving, J., Begthel, H., Peters, P.J., and Clevers, H. (2007). Identification of stem cells in small intestine and colon by marker gene Lgr5. Nature *449*, 1003–1007.

Cancer Genome Atlas Network (2012). Comprehensive molecular characterization of human colon and rectal cancer. Nature *487*, 330–337.

ENCODE Project Consortium (2012). An integrated encyclopedia of DNA elements in the human genome. Nature *489*, 57–74.

el Marjou, F., Janssen, K.P., Chang, B.H., Li, M., Hindie, V., Chan, L., Louvard, D., Chambon, P., Metzger, D., and Robine, S. (2004). Tissue-specific and inducible Cre-mediated recombination in the gut epithelium. Genesis *39*, 186–193.

Erpel, T., Superti-Furga, G., and Courtneidge, S.A. (1995). Mutational analysis of the Src SH3 domain: the same residues of the ligand binding surface are important for intra- and intermolecular interactions. EMBO J. *14*, 963–975.

Francavilla, C., Rigbolt, K.T., Emdal, K.B., Carraro, G., Vernet, E., Bekker-Jensen, D.B., Streicher, W., Wikström, M., Sundström, M., Bellusci, S., et al. (2013). Functional proteomics defines the molecular switch underlying FGF receptor trafficking and cellular outputs. Mol. Cell *51*, 707–722.

Giannakis, M., Hodis, E., Jasmine Mu, X., Yamauchi, M., Rosenbluh, J., Cibulskis, K., Saksena, G., Lawrence, M.S., Qian, Z.R., Nishihara, R., et al. (2014). RNF43 is frequently mutated in colorectal and endometrial cancers. Nat. Genet. *46*, 1264–1266.

Griffin, J.N., Del Viso, F., Duncan, A.R., Robson, A., Hwang, W., Kulkarni, S., Liu, K.J., and Khokha, M.K. (2018). RAPGEF5 regulates nuclear translocation of beta-catenin. Dev. Cell *44*, 248–260.e244.

Hatzis, P., van der Flier, L.G., van Driel, M.A., Guryev, V., Nielsen, F., Denissov, S., Nijman, I.J., Koster, J., Santo, E.E., Welboren, W., et al. (2008). Genome-wide pattern of TCF7L2/TCF4 chromatin occupancy in colorectal cancer cells. Mol. Cell. Biol. *28*, 2732–2744.

Henderson, B.R. (2000). Nuclear-cytoplasmic shuttling of APC regulates beta-catenin subcellular localization and turnover. Nat. Cell Biol. *2*, 653–660.

Ipsaro, J.J., Huang, L., and Mondragón, A. (2009). Structures of the spectrin-ankyrin interaction binding domains. Blood *113*, 5385–5393.

Jho, E.H., Zhang, T., Domon, C., Joo, C.K., Freund, J.N., and Costantini, F. (2002). Wnt/beta-catenin/Tcf signaling induces the transcription of Axin2, a negative regulator of the signaling pathway. Mol. Cell. Biol. *22*, 1172–1183.

Kim, Y.M., and Kim, D.H. (2013). dRAGging amino acid-mTORC1 signaling by SH3BP4. Mol. Cells *35*, 1–6.

Kim, Y.M., Stone, M., Hwang, T.H., Kim, Y.G., Dunlevy, J.R., Griffin, T.J., and Kim, D.H. (2012). SH3BP4 is a negative regulator of amino acid-Rag GTPase-mTORC1 signaling. Mol. Cell *46*, 833–846.

Koo, B.K., Spit, M., Jordens, I., Low, T.Y., Stange, D.E., van de Wetering, M., van Es, J.H., Mohammed, S., Heck, A.J., Maurice, M.M., and Clevers, H. (2012). Tumour suppressor RNF43 is a stem-cell E3 ligase that induces endocytosis of Wnt receptors. Nature *488*, 665–669.

Korinek, V., Barker, N., Morin, P.J., van Wichen, D., de Weger, R., Kinzler, K.W., Vogelstein, B., and Clevers, H. (1997). Constitutive transcriptional activation by a beta-catenin-Tcf complex in APC-/- colon carcinoma. Science *275*, 1784–1787.

Liu, G., Bafico, A., Harris, V.K., and Aaronson, S.A. (2003). A novel mechanism for Wnt activation of canonical signaling through the LRP6 receptor. Mol. Cell. Biol. *23*, 5825–5835.

Nicholson, A.M., Olpe, C., Hoyle, A., Thorsen, A.S., Rus, T., Colombe, M., Brunton-Sim, R., Kemp, R., Marks, K., Quirke, P., et al. (2018). Fixation and spread of somatic mutations in adult human colonic epithelium. Cell Stem Cell *22*, 909–918.e908.

Novellasdemunt, L., Foglizzo, V., Cuadrado, L., Antas, P., Kucharska, A., Encheva, V., Snijders, A.P., and Li, V.S.W. (2017). USP7 is a tumor-specific WNT activator for APC-mutated colorectal cancer by mediating β-catenin deubiquitination. Cell Rep. *21*, 612–627.

Ran, F.A., Hsu, P.D., Wright, J., Agarwala, V., Scott, D.A., and Zhang, F. (2013). Genome engineering using the CRISPR-Cas9 system. Nat. Protoc. *8*, 2281–2308.

Reed, J.C., Doctor, K.S., and Godzik, A. (2004). The domains of apoptosis: a genomics perspective. Sci. STKE *2004*, re9.

Rosin-Arbesfeld, R., Townsley, F., and Bienz, M. (2000). The APC tumour suppressor has a nuclear export function. Nature *406*, 1009–1012.

Sato, T., Vries, R.G., Snippert, H.J., van de Wetering, M., Barker, N., Stange, D.E., van Es, J.H., Abo, A., Kujala, P., Peters, P.J., and Clevers, H. (2009). Single Lgr5 stem cells build crypt-villus structures in vitro without a mesenchymal niche. Nature *459*, 262–265.

Sato, T., van Es, J.H., Snippert, H.J., Stange, D.E., Vries, R.G., van den Born, M., Barker, N., Shroyer, N.F., van de Wetering, M., and Clevers, H. (2011). Paneth cells constitute the niche for Lgr5 stem cells in intestinal crypts. Nature *469*, 415–418.

Schindelin, J., Arganda-Carreras, I., Frise, E., Kaynig, V., Longair, M., Pietzsch, T., Preibisch, S., Rueden, C., Saalfeld, S., Schmid, B., et al. (2012). Fiji: an open-source platform for biological-image analysis. Nat. Methods *9*, 676–682.

Shimomura, Y., Agalliu, D., Vonica, A., Luria, V., Wajid, M., Baumer, A., Belli, S., Petukhova, L., Schinzel, A., Brivanlou, A.H., et al. (2010). APCDD1 is a novel Wnt inhibitor mutated in hereditary hypotrichosis simplex. Nature *464*, 1043–1047.

Snippert, H.J., Schepers, A.G., van Es, J.H., Simons, B.D., and Clevers, H. (2014). Biased competition between Lgr5 intestinal stem cells driven by oncogenic mutation induces clonal expansion. EMBO Rep. *15*, 62–69.

Su, L.K., Kinzler, K.W., Vogelstein, B., Preisinger, A.C., Moser, A.R., Luongo, C., Gould, K.A., and Dove, W.F. (1992). Multiple intestinal neoplasia caused by a mutation in the murine homolog of the APC gene. Science *256*, 668–670.

Tosoni, D., Puri, C., Confalonieri, S., Salcini, A.E., De Camilli, P., Tacchetti, C., and Di Fiore, P.P. (2005). TTP specifically regulates the internalization of the transferrin receptor. Cell *123*, 875–888.

Wang, R., Wei, Z., Jin, H., Wu, H., Yu, C., Wen, W., Chan, L.N., Wen, Z., and Zhang, M. (2009). Autoinhibition of UNC5b revealed by the cytoplasmic domain structure of the receptor. Mol. Cell *33*, 692–703.

Yan, H.H.N., Lai, J.C.W., Ho, S.L., Leung, W.K., Law, W.L., Lee, J.F.Y., Chan, A.K.W., Tsui, W.Y., Chan, A.S.Y., Lee, B.C.H., et al. (2017). RNF43 germline and somatic mutation in serrated neoplasia pathway and its association with BRAF mutation. Gut *66*, 1645–1656.

STAR★METHODS

KEY RESOURCES TABLE

REAGENT or RESOURCE	SOURCE	IDENTIFIER
Antibodies		
α-Tubulin	Sigma-Aldrich	T9026
β-Actin	Sigma-Aldrich	A3854
β-catenin	BD	610154
β-catenin	Santa Cruz Biotechnology, Inc.	SC 7963
Active β-catenin	Millipore	05-665
ChromograninA	Abcam	ab15160
Cleaved Caspase3	R&D Biosciences	AF835
FLAG	Sigma-Aldrich	A2220
Lamin A	Abcam	ab8980
Lysozyme	DAKO	a0099
MYC	Santa Cruz Biotechnology, Inc.	764
phospho-S6	Cell Signaling Technology	2211
SH3BP4	Santa Cruz	393730
Tubulin	Sigma-Aldrich	T9026
Biological Samples		
Human intestinal blocks	University College London Hospital	http://www.uclh.nhs.uk/Pages/Home.aspx
Chemicals, Peptides, and Recombinant Proteins		
Tamoxifen	Sigma-Aldrich	T5648
Rapamycin	Sigma-Aldrich	R8781
5-ethynyl-2′deoxyuridine	Life Technologies	E10187
LF3	Sigma-Aldrich	SML1752
Critical Commercial Assays		
Dual-Luciferase-reporter assay system	Promega	E1910
In-Fusion® DH Cloning Kit	Takara	639650
RNAscope® 2.5 HD Reagent Kit—BROWN	Advanced Cell Diagnostics	322300
RNAscope® 2.5 HD Duplex Reagent Kit	Advanced Cell Diagnostics	322430
Experimental Models: Cell Lines		
HEK293T	ATCC	CRL-3216
SW480	ATCC	CCL-228
HEK293TΔSH3BP4	This paper	N/A
HEK293TΔAPC	Novellasdemunt et al., 2017	N/A
Experimental Models: Organisms/Strains		
Sh3bp4$^{tm1a(EUCOMM)Wtsi}$	Wellcome Trust Sanger Institute	N/A
Villin^{CreERT2}	el Marjou et al., 2004	N/A
Apc^{min}	Su et al., 1992	N/A
Oligonucleotides		
Primer sequence, see Table S1	This paper	N/A
Recombinant DNA		
Plasmid: PX459	Ran et al., 2013	Addgene plasmid #62988
pcDNA_SH3BP4	This paper	N/A
pcDNA_ SH3BP4ΔN	This paper	N/A
pcDNA_ SH3BP4W92A	This paper	N/A

(*Continued on next page*)

Continued		
REAGENT or RESOURCE	SOURCE	IDENTIFIER
pcDNA_ SH3BP4ΔSH3	This paper	N/A
pcDNA_ SH3BP4ΔZU5	This paper	N/A
pcDNA_ SH3BP4ΔDD	This paper	N/A
Other		
RNAScope probe *Lgr5*	Advanced Cell Diagnostics	ref #312171
RNAScope probe *Olfm4*	Advanced Cell Diagnostics	ref #311831
RNAScope probe *Axin2*	Advanced Cell Diagnostics	ref #400338
RNAScope probe *Sh3bp4*	Advanced Cell Diagnostics	ref #474731

CONTACT FOR REAGENT AND RESOURCE SHARING

Further information and requests for reagents may be directed to and will be fulfilled by the Lead Contact, Vivian Li (vivian.li@crick.ac.uk).

EXPERIMENTAL MODEL AND SUBJECT DETAILS

Animals

All animal maintenance and regulated procedures were carried out according to Project License constraints (70/8560) and Home Office guidelines and regulations. In accordance with the 3Rs, the smallest sample size was chosen that could give a significant difference. *Sh3bp4*$^{fl/fl}$ mouse was obtained from the International Mouse Strain Resource generated by the Wellcome Trust Sanger Institute (*Sh3bp4*$^{tm1a(EUCOMM)Wtsi}$), where two loxP sites were inserted flanking the critical exon 4. *Sh3bp4*$^{fl/fl}$ mice were crossed to *Villin*^{CreERT2} (el Marjou et al., 2004) or *Apc*^{min} (Su et al., 1992). Animals of both sexes at age 6-7 weeks were used for the different experimental conditions and harvested as indicated.

Tamoxifen was injected intraperitoneally for 3 consecutive days (1.5mg/10 g of mouse weight) from a 20mg/ml stock solution. 5-ethynyl-2′deoxyuridine (EdU) (Life Technologies) was injected intraperitoneally (0.3mg/10 g of mouse weight) from a 10mg/ml stock solution. Rapamycin was injected intraperitoneally 60 days after the first tamoxifen injections. Mice were injected every other day for 15 consecutive times with 10mg.Kg^{-1} of rapamycin. Rapamycin solution was prepared in ethanol at 50mg/ml and diluted 5% Tween-80, 5% PEG400 in PBS to a final concentration of 2mg/ml.

METHOD DETAILS

Cell culture, transfection and TOPFlash assay

Cell lines were maintained in DMEM GlutaMAX (GIBCO) supplemented with 5% fetal bovine serum (FBS) (GIBCO) and 100 units/ml penicillin (GIBCO) and 100 μg/ml streptomycin (GIBCO). HEK293TΔAPC was generated previously by CRISPR targeting with truncation at 1225a.a (APC4) (Novellasdemunt et al., 2017). All cell lines were incubated in a humidified atmosphere of 5% CO2 at 37°C. Cells were seeded in plates 24hrs before transfection and plasmids were transfected using polyethylenimine (Polysciences) according to the manufacturer's instructions. For the TOPFlash luciferase assay, cells were seeded at a density of 1x10^5 cells/well in a 24-well plate. The cells were then transfected with 200ng of TOPFlash or FopFlash plasmid constructs (Korinek et al., 1997). Transfection efficiency was normalized against the co-transfected renilla luciferase activity (10ng/well). Wnt3A-conditioned medium was added to the cells 24hrs post-transfection. Treated cells were lysed after 16hrs using luciferase lysis buffer (Promega), and luciferase activity was measured using the Dual-Luciferase-reporter assay system (Promega) and analyzed in the microplate luminometer (Centro XS3 LB960, Berthold Technologies).

For LF3 inhibitor treatment, cells were seeded in plates followed by treatment with Wnt3a+Rspondin-conditioned media or control media. 16hr later, LF3 inhibitor treatment (30 uM) or DMSO was added to the media for an additional 8 hr.

Crypts/Villi fractionation and organoid culture

Small intestine was washed with cold PBS and cut into small pieces. Sequential incubations with 1mM EDTA for 20min at 4°C were performed. The resulting fractions of crypts and villi (in increasing purities) were passed through a 70μm cell strainer each time. Fractions from above and below the strainer were collected and checked under the microscope for purity. Fractions of similar purity were combined for RNA extraction, organoid culture or crypt cell sorting. For organoid culture, crypts were seeded in 20 μl of Cultrex® BME Type 2 RGF PathClear (Amsbio, 3533-010-02) in individual wells of a 24-well plate and cultured as previously described (Sato et al., 2009). *Apc* mutant organoids (ΔAPC) was previously generated by CRIPSR targeting with truncation at 680aa (*Apc5*) (Novellasdemunt et al., 2017).For sorting experiments, isolated crypts from *Lgr5*-GFP mice(Barker et al., 2007) were incubated in

trypsin for 20 min at 37°C, followed by trituration with a glass pipette. Dissociated cells were passed through cell strainer with a pore size of 20 μm. GFPhi, GFPlow cells were sorted by flow cytometry. Unviable epithelial cells were determined by positive staining for propidium iodide.

Plasmids and reagents

Full-length *SH3BP4* was amplified by PCR from HEK293T cell cDNA. Briefly, 50ng of cDNA was amplified using Phusion® High-Fidelity PCR Master Mix (Biolabs). PCR products were cloned into pcDNA-FLAG plasmids using the In-Fusion® DH Cloning Kit, according to the manufacturer's instructions. The SH3BP4 dead domain constructs were generated using the In-Fusion® DH Cloning Kit with primers specifically designed for each domain. Each primer contained a homology arm of 15 base pairs (bp). Primers sequences are shown in Key Resources Table.

The constructs with site directed mutagenesis were generated by PCR of the original construct with the indicated mutagenic primers Phusion® High-Fidelity PCR Master Mix was used and non-mutated parental DNA template was digested with the restriction endonuclease *DpnI*.

CRISPR/Cas9 genome engineering

To generate SH3BP4 knock-out HEK293T cells, single guide RNA (sgRNA) was designed for specific target regions, as previously described (Ran et al., 2013). HEK293T cells were transfected with plasmids encoding Cas9 and sgRNAs (PX459, #62988, Addgene, a gift from Feng Zhang lab). SH3BP4 was targeted using the gRNA: 5′gggcgaccatctctacgtct3′. 48hrs after transfection, cells were selected using 2μg/ml puromycin. Single, puromycin-resistant cells were selected and expanded for genomic DNA extraction. The targeted locus was amplified and subcloned into a TA-cloning vector for cloning sequencing. Indel mutations were confirmed by sequencing and loss of protein by western blot analysis.

Antibodies and western blot analysis

Cells were lysed in cold lysis buffer containing 150 mM NaCl, 30 mM Tris (pH 7.5), 1 mM EDTA, 1% Triton X-100, 10% Glycerol, 0.1 mM PMSF (phenylmethylsulfonyl fluoride), 0.5 mM DTT (dithiothreitol), protease inhibitor cocktail tablets (EDTA-free) (Roche), and phosphatase inhibitor cocktail tablets (Roche). Lysates were pelleted for 30 min at 13200 rpm and supernatants kept for protein quantification (Bradford assay). Equal amounts of cellular protein were resolved in 10% sodium dodecyl sulfate–polyacrylamide gels (SDS-PAGE) and subsequently transferred to polyvinylidene difluoride (PVDF) membranes. Membranes were blocked using 5% milk (OXOID) or 5% bovine serum (BSA) (Sigma) for phosphorylated proteins immunoblots, in Tris-buffered saline TBS (50mM Tris, 150mM NaCl, pH7.6) containing 0.1% Tween-20 (Sigma) (TBST) for 1 hour, and primary antibodies were added in blocking solution. The following antibodies were used: Active β-catenin (1:1000, Millipore 05-665), β-catenin (1:1000, BD 610154), SH3BP4 (1:500, Santa Cruz 393730), FLAG (1:1000, Sigma A2220), β-Actin (1:25000 Sigma A3854), Lamin A (1:1000, ab8980), Tubulin (1:5000, T9026). Primary antibody incubations were carried out at 4°C overnight. After washing with TBST, the appropriate HRP-conjugated secondary was added (1:5000 in blocking buffer) for 2 hours at room temperature. Antibody binding was detected using chemiluminescence ECL Prime Western Blotting Substrate (GE Healthcare).

Real-time quantitative RT-PCR

RNA was extracted according to the manufacturer's instructions (QIAGEN RNAeasy). cDNA was prepared using Maxima first strand cDNA synthesis kit (#1672, Thermo Scientific). Quantitative PCR detection was performed using iTaq SYBR Green Supermix. The reaction mixture without template cDNA was run as a control. Expression was normalized to *ACTIN* as indicated and data were expressed as mean ± standard error. Primers sequences are indicated in Key Resource Table.

Immunofluorescence

Cells were grown on poly-L-lysine-coated (Sigma) glass coverslips in 12-well, fixed with 4% paraformaldehyde (PFA) for 15 min, and permeabilised using 0.5% Triton X-100 in PBS for 10 min. Cells were blocked with 1% BSA in PBS for 1h before overnight incubation with β-catenin (1:1000, BD 610154) and FLAG (1:1000, Sigma F7425) at 4°C. Cells were washed three times with PBS and incubated with secondary antibodies conjugated to Alexa Fluor 488 or 568 at room temperature for 1h in the dark. Cells were washed three times with PBS and stained with DAPI for 10 min. Coverslips were washed another three times with PBS and were then mounted with Aqua Poly/Mount (Polysciences). Images were taken using a Leica SPE confocal microscope. Each fluorophore was imaged separately using 405, 488 and 561 channels. Confocal images were taken as Z stacks and processed using Fiji (Schindelin et al., 2012).

Histology and Immunohistochemistry

Small intestine and colon tissues were fixed in 10% buffered formaldehyde for 16hrs time and embedded in paraffin. For staining, 4μm sections were de-paraffinized using xylene and rehydrated through a graded series of ethanol. Antigen retrieval was performed for 20 min at high temperature in either 0.01M citrate buffer (pH6) or Tris-EDTA (10mM Tris base, 1mM EDTA solution, pH9), depending on the antibody. The following antibodies were used: Lysozyme (1:1500, DAKO a0099), Cleaved Caspase3 (1:900, RD AF835), phospho-S6 (1:400, CS 2211), MYC (1:1500, 10828-1-AP), ChromograninA (1:1250, ab15160), β-catenin (1:4000, SC 7963), SH3BP4

(1:100, SC393730). Samples were blocked using 1% BSA and incubated overnight with the desired antibody or negative control at 4°C. Finally, slides were incubated with the secondary antibody for 1h and washed three times with PBS. For colorimetric staining with diaminobenzidine (DAB) slides were incubated with peroxidase substrate and mounted. Mice adenomas were graded by analysis of H&E stained sections by pathologist as follow: low grade dysplasia: mildly distorted glandular structures, branching villi and tubular crypt proliferation, mild nuclear and cellular atypism, and intact basement membrane; high grade dysplasia: moderately or severely distorted glandular structures with branching villi, severe nuclear and cellular atypism, increased mitotic figures, increased atypical mucous retention. The human CRC sample was provided by the University College London Hospital. Ethical approval was obtained from the UK Research Ethics Committee and informed consent was obtained from subjects.

RNAScope *in situ* hybridization

In situ hybridization (ISH) for *Lgr5, Olfm4, Axin2* and *Sh3bp4* was performed using the RNAscope FFPE assay kit (Advanced Cell Diagnostics, Inc., Hayward, CA, USA) according to the manufacturer's instructions. Briefly, 4μm formalin-fixed, paraffin-embedded tissue sections were pre-treated with heat and protease digestion before hybridization with a target probe. Thereafter, an HRP-based signal amplification system was hybridized to the target probes (*Lgr5* ref #312171, *Olfm4* ref #311831, *Axin2* ref #400338, *Sh3bp4* ref #474731) before color development with 3,3′-diaminobenzeidine tetrahydrochloride (DAB). *Lgr5* staining quantification in Apc^{min} adenomas was performed with the Segmentation Macro from ImageJ.

QUANTIFICATION AND STATISTICAL ANALYSIS

Statistical analyzes were performed using GraphPad Prism8 software. Normal distribution of data was determined using the D'Agostino and Pearson omnibus test. For parametric data, statistical significance was determined using a student's unpaired, two-tailed t test. In cases where more than two groups were being compared, then a one-way ANOVA was used. In instances where the N was too small to determine normal distribution, or the data were non-parametric, a two-tailed Mann-Whitney U-test was used. P values are represented as ***$p \leq 0.001$, **$p \leq 0.01$, *$p \leq 0.05$, non-significant (ns- $p > 0.05$).

Cell Reports, Volume *26*

Supplemental Information

SH3BP4 Regulates Intestinal Stem Cells and Tumorigenesis by Modulating β-Catenin Nuclear Localization

Pedro Antas, Laura Novellasdemunt, Anna Kucharska, Isobel Massie, Joana Carvalho, Dahmane Oukrif, Emma Nye, Marco Novelli, and Vivian S.W. Li

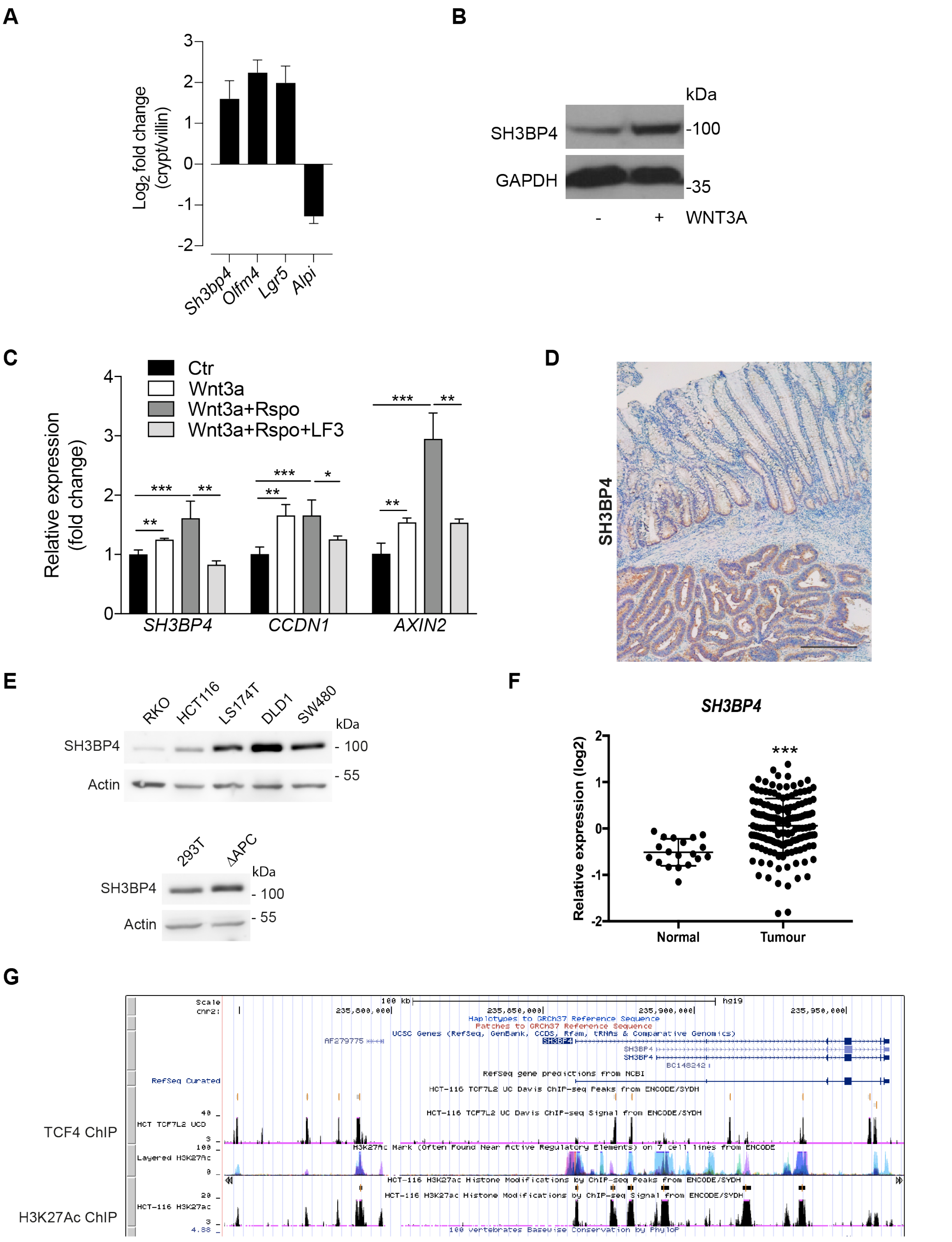

Figure S1. SH3BP4 is expressed at the crypt and is modulated by Wnt, Related to Figure 1.
(A) RT-qPCR analysis of the indicated genes in crypt versus villus fractions. Data represent average ±SD from 3 independent experiments. (B) Western blot analysis of SH3BP4 protein levels in HEK293T cells stimulated with WNT3A condition media for 16hr (n=3). (C) RT-qPCR of *SH3BP4* and Wnt target genes *AXIN2*, *CCND1* in the indicated treatment conditions (16hr). Expression data are presented as fold induction ±SD normalised to β-actin (n=3). (D) Representative immunohistochemistry staining of SH3BP4 in human CRC tissue. Scale bar, 100 μm. (E) Western blot analysis showing SH3BP4 expression in different colorectal cancer cell lines (upper), and in HEK293T and ΔAPC cells (APC4 cells from (Novellasdemunt et al., 2017)) (n=3). (F) mRNA expression analysis of SH3BP4 in 173 CRC patients (data obtained from the Cancer Genome Atlas) (TCGA, 2012). (G) TCF4-binding sites are found upstream and throughout the *SH3BP4* gene locus, and are co-localized with the H3K27Ac peaks. Data obtained from ENCODE where TCF4 ChIP-seq was performed in HCT116 cells (GSM782123) (Consortium, 2012).

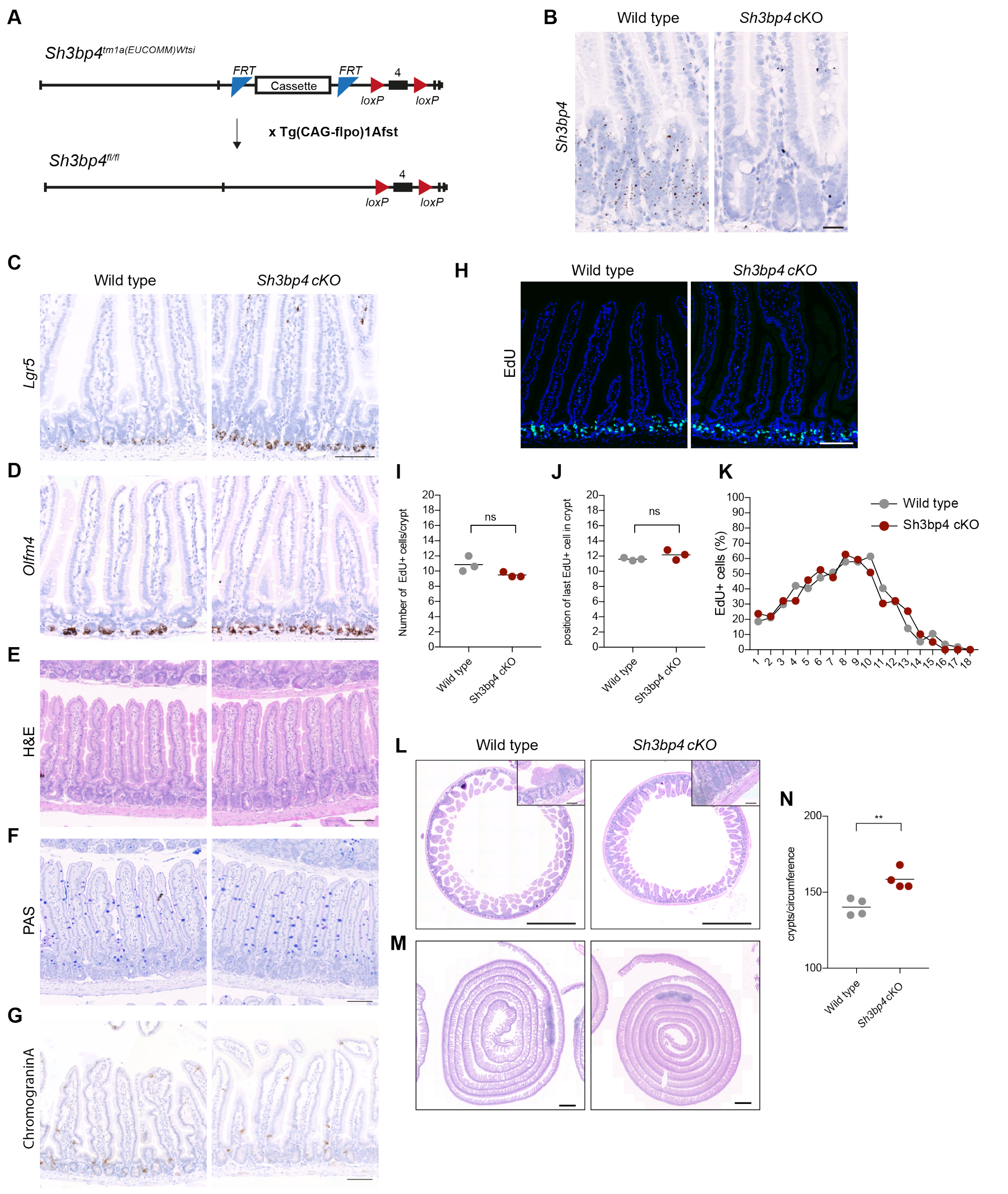

Figure S2. Characterization of *Sh3bp4* cKO intestine 3 months (A-B) or 25 days (C-K) after deletion, Related to Figure 2. (A) Schematic overview of Sh3bp4 targeting strategy. *Sh3bp4$^{tm1a(EUCOMM)Wtsi}$* mouse was first crossed to Tg(CAG-Flpo)1Afst strain to remove the lacZ-neomycin cassette. This results in the conditional *Sh3bp4$^{fl/fl}$* strain flanking exon 4. (B) RNAScope ISH showing efficient Cre-mediated *Sh3bp4* deletion in the intestine after tamoxifen induction. Scale bar, 100 µm. (C-D) Representative image of RNAscope ISH of ISC marker, *Lgr5* (C) and *Olfm4* (D), 3 months after *Sh3bp4* loss. (E-G) Tissues stained with H&E (E), periodic acid Schiff (PAS, goblet cells) (F), and chromogranin A (enteroendocrine cells) (G) showed no difference between wild-type and *Sh3bp4* cKO intestine. Scale bar, 100 µm. (H) Immunofluorescent staining of EdU (green) and DAPI (blue). (I-K) Quantitation of EdU+ proliferating cells shows no differences in number of EdU^{+} cells per crypt (I), nor in the height of the proliferative zone (J), nor in the distribution of the proliferative cells along the crypt-villus axis (K). (L-M) H&E staining of wild-type and *Sh3bp4* cKO intestine in transversal (L) and frontal (M) intestinal sections showing more compact crypt-villus units in *Sh3bp4* cKO compared to wild-type. Scale bar, 1mm; inset 100 µm. (N) Quantitation of number of crypts per circumference in the small intestine. Each dot represents the average number of crypts counted in 3 different transversal cuts of the jejunum per mouse. n=4 animals/group. **P≤ 0.01.

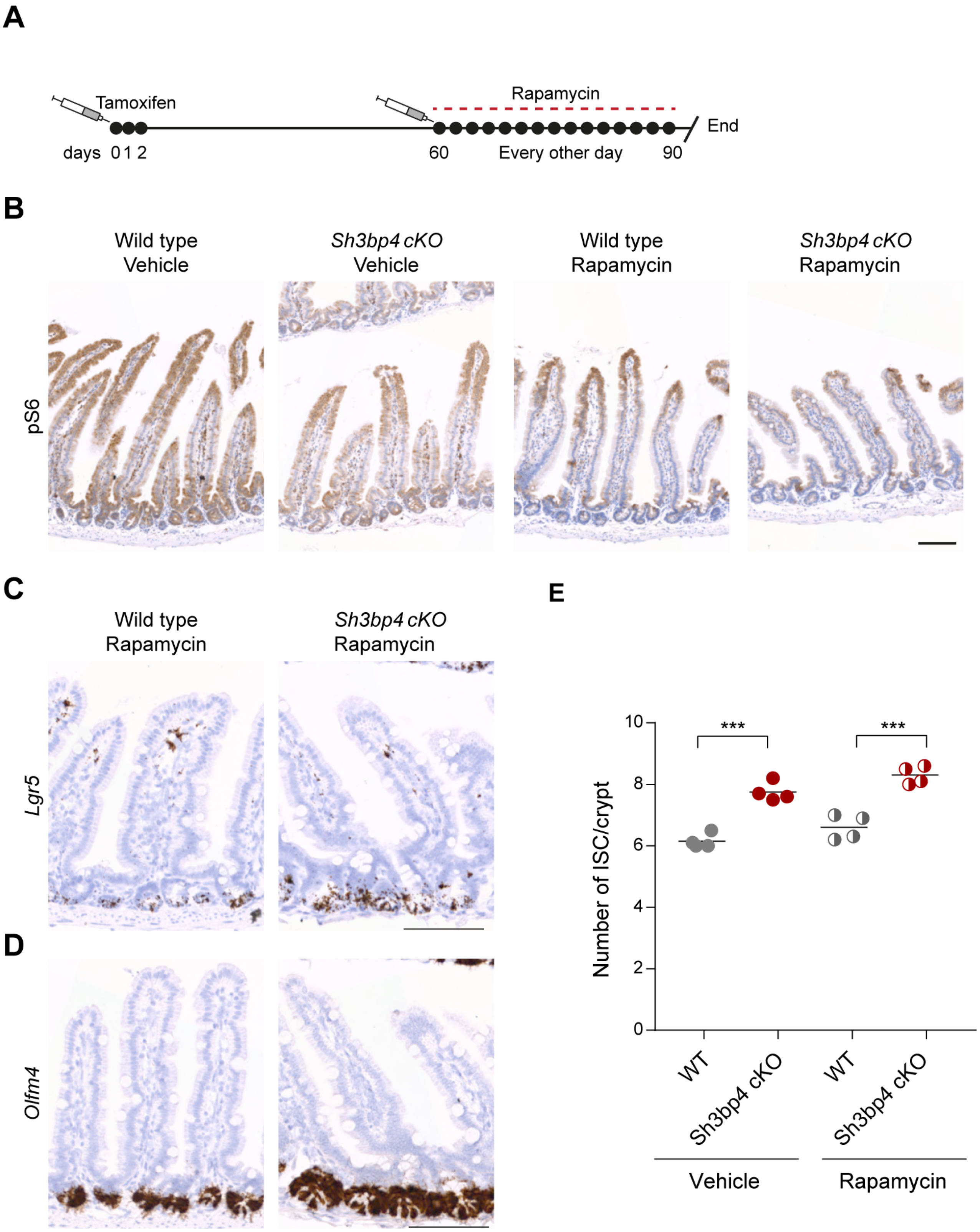

Figure S3. Increased number of stem cells in *Sh3bp4* cKO mice is independent of mTOR pathway, Related to Figure 2. (A) Schematic representation of rapamycin treatment strategy. *Sh3bp4* deletion was induced at 6 weeks-old by tamoxifen administration, and rapamycin treatment started 2 months after with intraperitoneal injections every other day for a month, after which mice were sampled. (B) Representative immunohistochemistry of phospho-RSP6 (pS6) showing reduction of RSP6 phosphorylation in animals treated with rapamycin for a month. (C-D) Representative images of RNAscope ISH of the ISC marker *Lgr5* (C) and *Olfm4* (D). Scale bar, 100µm. (E) Quantitation of number of *Olfm4*+ ISCs per crypt shows that mTOR inhibition has no effect on the increased number of ISCs observed in *Sh3bp4* cKO compared to wild-type. Each dot represents the average number of *Olfm4*+ cells per crypt per mouse (determined from at least 30 crypts analyzed). n=4 animals/group.

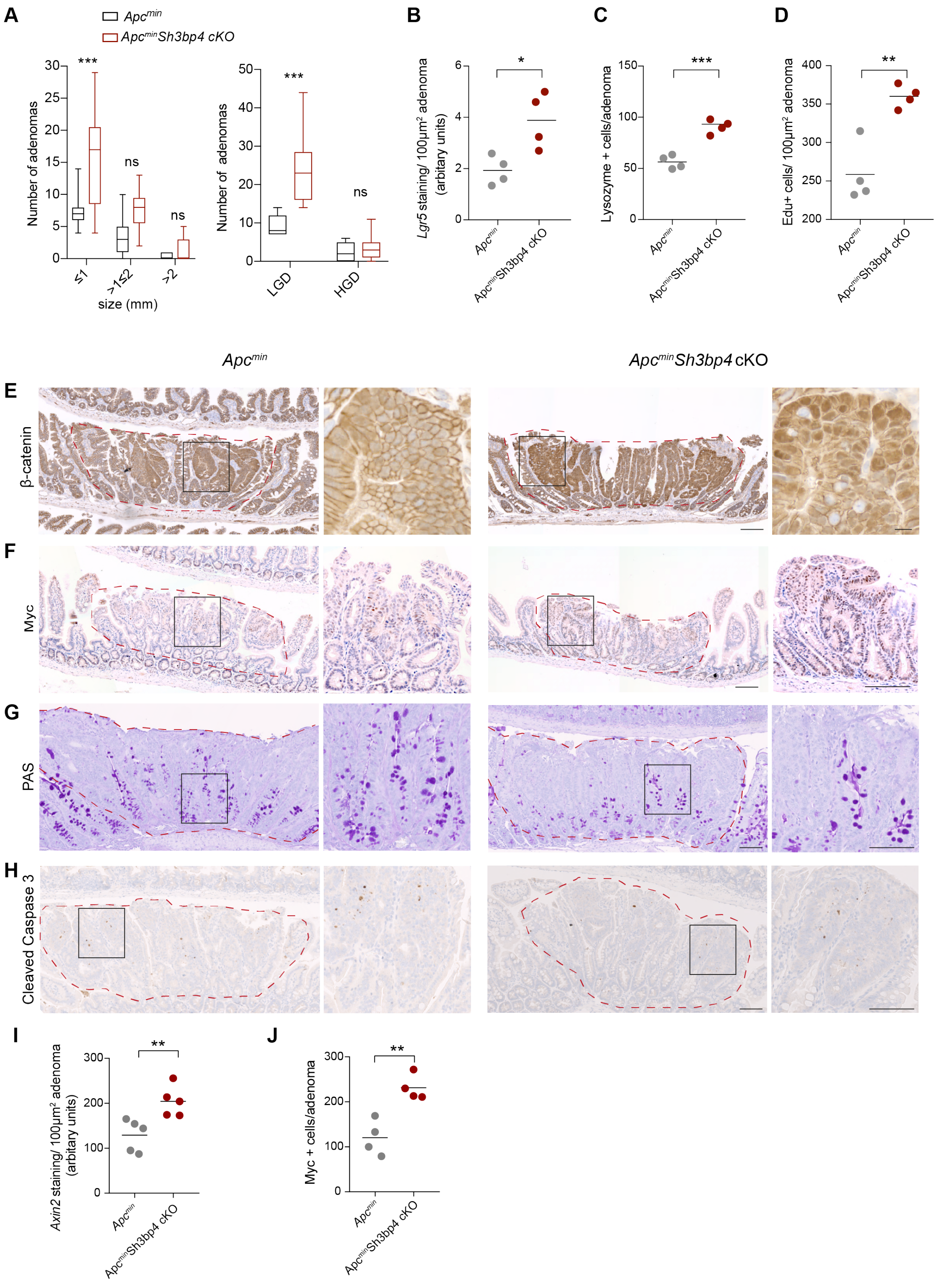

Figure S4. Increased Wnt/β-catenin signaling and reduced differentiation in *Apc^min^Sh3bp4* cKO adenomas, Related to Figure 3. (A) Distribution of adenomas categorised based on size (left) and grade of dysplasia (right). LGD, low grade dysplasia; HGD, high grade dysplasia. The adenoma classification as LGD and HGD are detailed in the Methods section. Data are represented as mean ±SD. ***P≤ 0.001, ns, non-significant. (B) Quantification of *Lgr5* staining per 100µm^2 adenoma in Figure 3D. (C) Quantification of Lysozyme$^+$ Paneth cells in Figure 3F. (D) Quantification of EdU+ proliferative cells in *Apc^{min}* and *Apc^{min}Sh3bp4* cKO adenomas in Figure 3G. Each dot represents the average of at least 5 adenomas (with similar size and grade of dysplasia) per animal. Black bar indicates the mean per group. n=4 animals/group. ***P≤ 0.001, **P≤ 0.01, *P≤0.05. (E-H) Immunohistochemistry staining of

β-catenin (E), Myc (F), PAS (G) and cleaved-caspase 3 (H) in *Apc*^{min} and *Apc*^{min}*Sh3bp4* cKO intestine. Increased nuclear β-catenin and Myc expression is observed in *Apc*^{min}*Sh3bp4* cKO adenomas compared to WT. Magnifications of the boxed area are shown. PAS staining shows reduced number of Goblet cells in adenomas with *Sh3bp4* deletion. Cleaved-caspase 3 staining show similar levels of apoptosis in *Apc*^{min} and *Apc*^{min}*Sh3bp4* cKO adenomas. Images are representative of at least 6 animals analyzed per group. Scale bar, 100µm. (I) Quantification of *Axin2* staining per 100□m^2 adenoma in Figure 3E. Each dot represents the average of at least 5 adenomas (with similar size and grade of dysplasia) per animal. Black bar indicates the mean per group. n=5 animals/group. (J) Quantification of Myc+ cells in *Apc*^{min} and *Apc*^{min}*Sh3bp4* cKO adenomas in Figure S4F. Each dot represents the average of at least 5 adenomas (with similar size and grade of dysplasia) per animal. Black bar indicates the mean per group. n=4 animals/group. **$P \leq 0.01$.

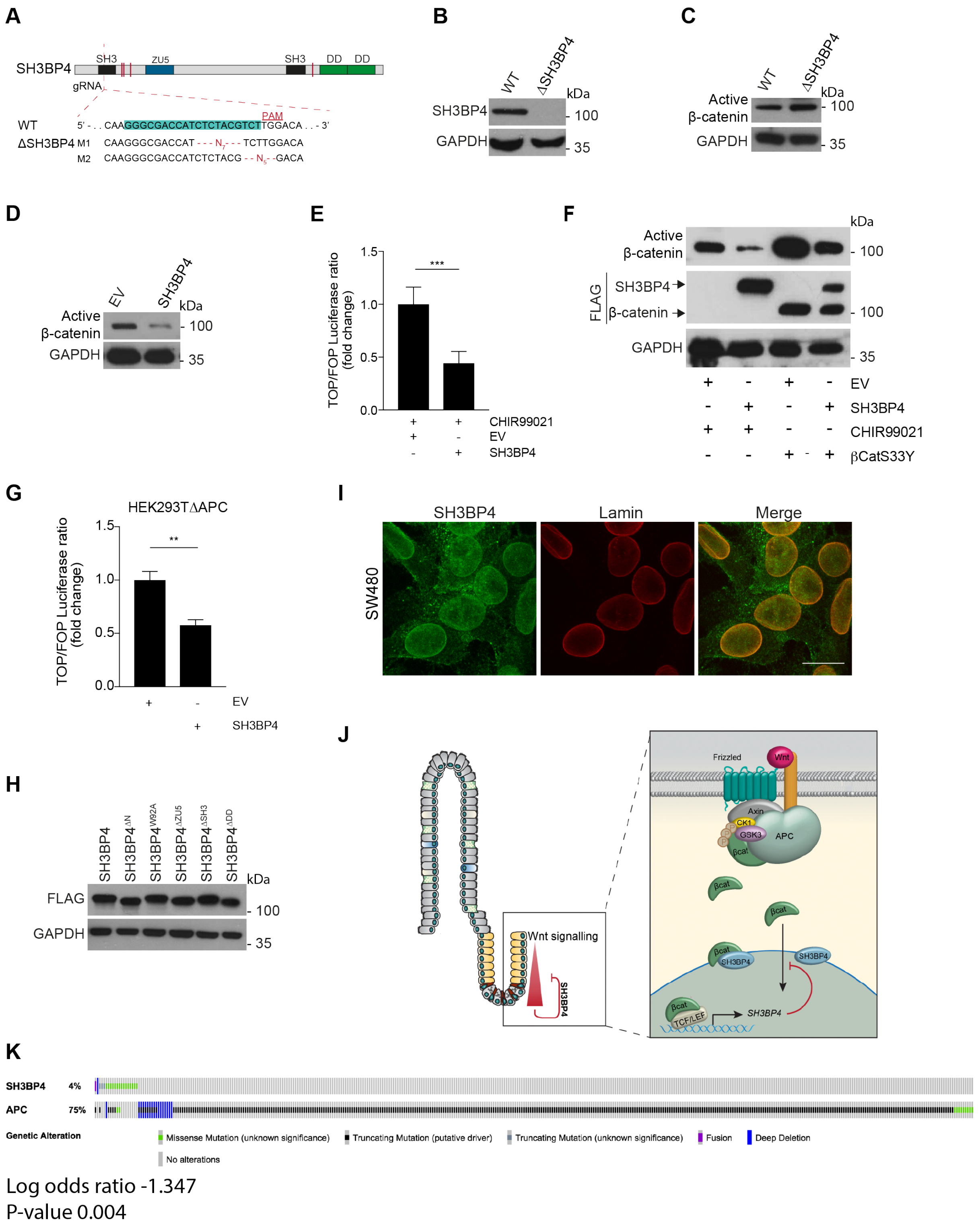

Figure S5. SH3BP4 negatively regulates Wnt signaling, Related to Figure 4. (A) Schematic representation of the CRISPR/Cas9 targeting strategy of *SH3BP4*. Genotyping of the selected ΔSH3BP4 mutant clone reveals homozygous frameshift deletions on both alleles, indicated as M1 and M2. A 7bp- and a 5bp deletion were retrieved from the ΔSH3BP4 clone, resulting in protein truncation at 110 and 89 amino acids respectively. (B) Immunoblot analysis showing protein loss in the ΔSH3BP4 mutant clone. (C) ΔSH3BP4 cells show increased levels of active-β-catenin in Wnt3A-induced HEK293T cells when compared to wild-type. (D) Ectopic expression of SH3BP4 in HEK293T cells shows reduced levels of active-β-catenin. (E) HEK293T cells overexpressing EV or SH3BP4 plasmids were treated with 2µM of GSK3 inhibitor CHIR99021 for 24 hours and relative TOPFlash reporter activity was measured. (F) Western blot shows that SH3BP4 reduces the active β-catenin levels induced by GSK3 inhibition (CHIR99021) and β-cateninS33Y expression. (G) SH3BP4 expression suppresses TOPFlash reporter activity induced by APC truncation (HEK293T ΔAPC) (Novellasdemunt et al., 2017). (H) Western blot analysis showing similar protein expression level among different FLAG-tagged SH3BP4 WT and mutant constructs. (I) Immunofluorescence of SH3BP4 (green) and the perinuclear marker Lamin (red) in SW480 cells. Scale bar, 100 µm. (J) Proposed model for the negative feedback

role of SH3BP4 in Wnt signaling. SH3BP4 is expressed at the Wnt-active stem cell compartment as a negative feedback mechanism to control Wnt/β-catenin signaling and maintain ISC homeostasis. SH3BP4 is present at the perinuclear membrane to restrict β-catenin nuclear translocation.

Table S1. Primer sequences, Related to STAR Methods.

REAGENT or RESOURCE	SOURCE	IDENTIFIER
Oligonucleotides		
SH3BP4, F: 5’ gtcaaggtgtaggagagggagg 3’, R: 5’ggcaaaggactcagaggaatg3’	This paper	N/A
SH3BP4$^{\Delta N}$ F: 5’gtggcggccgctcgaggccatgcccttgaactaccggaactcaacac3’; R: 5’ggcaaaggactcagaggaatg 3’	This paper	N/A
SH3BP4$^{\Delta DD}$ F:5’ gtcaaggtgtaggagagggagg3’ R:5’ tccttgtagtctcgagcctccagcagcaccgaggtgctca3’;	This paper	N/A
SH3BP4$^{\Delta SH3}$ F: 5’gtgtccagcctcaagctggtggtcggcagggcccgg3’ R: 5’cctgccgaccaccagttgaggctggacaccgggcgg3’	This paper	N/A
SH3BP4$^{\Delta ZU5}$ F 5’cagacccaagccgtgctttttagcaaaagcacagtg3’ R: 5’gcttttgctaaaaagcacggcttgggtctggcccca3’	This paper	N/A
SH3BP4^{W92A} F: 5’ cacatctggcggtgaggcgtggtacgcacacaac 3’, R: 5’ gttgtgtgcgtaccacgcctcaccgccagatgtg	This paper	N/A
RT-qPCR *AXIN2*, F: 5’ agtgtgaggtccacggaaac3’, R: 5’ cttcacactgcgatgcattt 3’	This paper	N/A
RT-qPCR *MYC* F: 5’gtcgtagtcgaggtcatag3’, R: 5’ tctccttgcagctgcttag3’	This paper	N/A
RT-qPCR *CCND1* F: 5’aaatatcccgacagggttcc3’, R: 5’gtgttctctccgctgtaggc3’	This paper	N/A
RT-qPCR *Sh3bp4* F: 5’gtggcgtccgtcctagaaaa3’, R: 5’gactcgtaggcgtccatctg3’	This paper	N/A
RT-qPCR *ACTIN* F: 5’gagcgcggctacagctt3’, R: 5’tccttaatgtcacgcacgattt3’	This paper	N/A
SH3BP4 CRISPR/Cas9 gRNA: 5’gggcgaccatctctacgtct3’	This paper	N/A
